# Definition of the estrogen negative feedback pathway controlling the GnRH pulse generator in female mice

**DOI:** 10.1038/s41467-022-35243-z

**Published:** 2022-12-02

**Authors:** H. James McQuillan, Jenny Clarkson, Alexia Kauff, Su Young Han, Siew Hoong Yip, Isaiah Cheong, Robert Porteous, Alison K. Heather, Allan E. Herbison

**Affiliations:** 1 Centre for Neuroendocrinology, University of Otago School of Biomedical Sciences, Dunedin, 9054 New Zealand; 2 Department of Physiology, University of Otago School of Biomedical Sciences, Dunedin, 9054 New Zealand; 3 Department of Physiology, Development and Neuroscience, University of Cambridge, Cambridge, CB2 3EG UK

**Keywords:** Infertility, Cellular neuroscience

## Abstract

The mechanisms underlying the homeostatic estrogen negative feedback pathway central to mammalian fertility have remained unresolved. Direct measurement of gonadotropin-releasing hormone (GnRH) pulse generator activity in freely behaving mice with GCaMP photometry demonstrated striking estradiol-dependent plasticity in the frequency, duration, amplitude, and profile of pulse generator synchronization events. Mice with Cre-dependent deletion of ESR1 from all kisspeptin neurons exhibited pulse generator activity identical to that of ovariectomized wild-type mice. An in vivo CRISPR-Cas9 approach was used to knockdown ESR1 expression selectively in arcuate nucleus (ARN) kisspeptin neurons. Mice with >80% deletion of ESR1 in ARN kisspeptin neurons exhibited the ovariectomized pattern of GnRH pulse generator activity and high frequency LH pulses but with very low amplitude due to reduced responsiveness of the pituitary. Together, these studies demonstrate that estrogen utilizes ESR1 in ARN kisspeptin neurons to achieve estrogen negative feedback of the GnRH pulse generator in mice.

## Abstract (web summary)

Estrogen secreted by the ovary controls how the brain drives pulsatile reproductive hormone secretion. The authors show that in mice, estrogen receptor alpha within a specific population of hypothalamic kisspeptin neurons is the principal pathway through which estrogen brings about this classic negative feedback pathway common to all mammals.

## Introduction

Estrogen negative feedback represents one of the classic homeostatic mechanisms operating in vertebrates to control fertility. Reflecting ovarian status, circulating 17-β-estradiol modulates the brain and pituitary gland to control the pulsatile pattern of gonadotropin secretion. Although it has been known for over 50 years that gonadectomy dramatically increases the frequency and amplitude of gonadotrophin pulses^1^, the mechanisms underlying estrogen negative feedback have remained elusive with numerous hypothalamic brain regions, neuronal phenotypes, and intracellular signaling pathways being implicated^2–4^. One rare consensus has been that estrogen receptor alpha (ESR1) is the key receptor underlying estrogen negative feedback^3,5^.

A favored current hypothesis is that estradiol acts through the arcuate nucleus kisspeptin (ARN^KISS^) neurons to bring about estrogen negative feedback. This population of neurons operates as the gonadotropin-releasing hormone (GnRH) pulse generator^6–8^, expresses ESR1^9^ and has its kisspeptin biosynthesis strongly regulated by estradiol in multiple species^9–12^. Thus, as the GnRH pulse generator, the ESR1-expressing ARN^KISS^ neurons would appear to be an ideal direct target for circulating estradiol to suppress pulsatile GnRH secretion^9^. However, in marked contrast to *Kiss1* mRNA expression, assessments of ARN^KISS^ neuron activity and function have not supported this hypothesis. Critically, estrogen negative feedback of luteinizing hormone (LH) secretion remains present in adult mice with ESR1 deleted selectively from kisspeptin neurons^13,14^ as well as in adult rats with toxin-induced ablation of ARN^KISS^ neurons^15^. Similarly, an acute ARN^KISS^ neuron-selective knockdown of ESR1 was not found to have any effect on LH secretion^16^. At a cellular level, electrophysiological studies have found little evidence for gonadal manipulation or selective ESR1 deletion to have any substantial or consistent effects on the firing rates of ARN^KISS^ neurons in vitro^17–21^.

A significant long-standing problem when examining the neural mechanism of estrogen negative feedback in vivo is that effects in the brain are often obscured by independent, parallel actions of estradiol at the pituitary gland. Monitoring the activity of the GnRH pulse generator directly in freely behaving mice with GCaMP fiber photometry overcomes this issue. Using this approach, combined with traditional genetic, viral, and acute CRISPR-mediated gene editing methodologies, we demonstrate that ESR1 in the ARN^KISS^ neurons is responsible for virtually all of the suppressive effects of estradiol on pulsatile LH secretion in the brain. In contrast to prior hypotheses regarding likely multi-modal mechanisms of estrogen action within the brain to suppress GnRH secretion^3,4,22,23^, the present study demonstrates that the negative feedback pathway in mice is primarily dependent on a single receptor in a single neuronal phenotype.

## Results

### ARN^KISS^ neuron pulse generator activity increases following ovariectomy

**Fig. 1:**
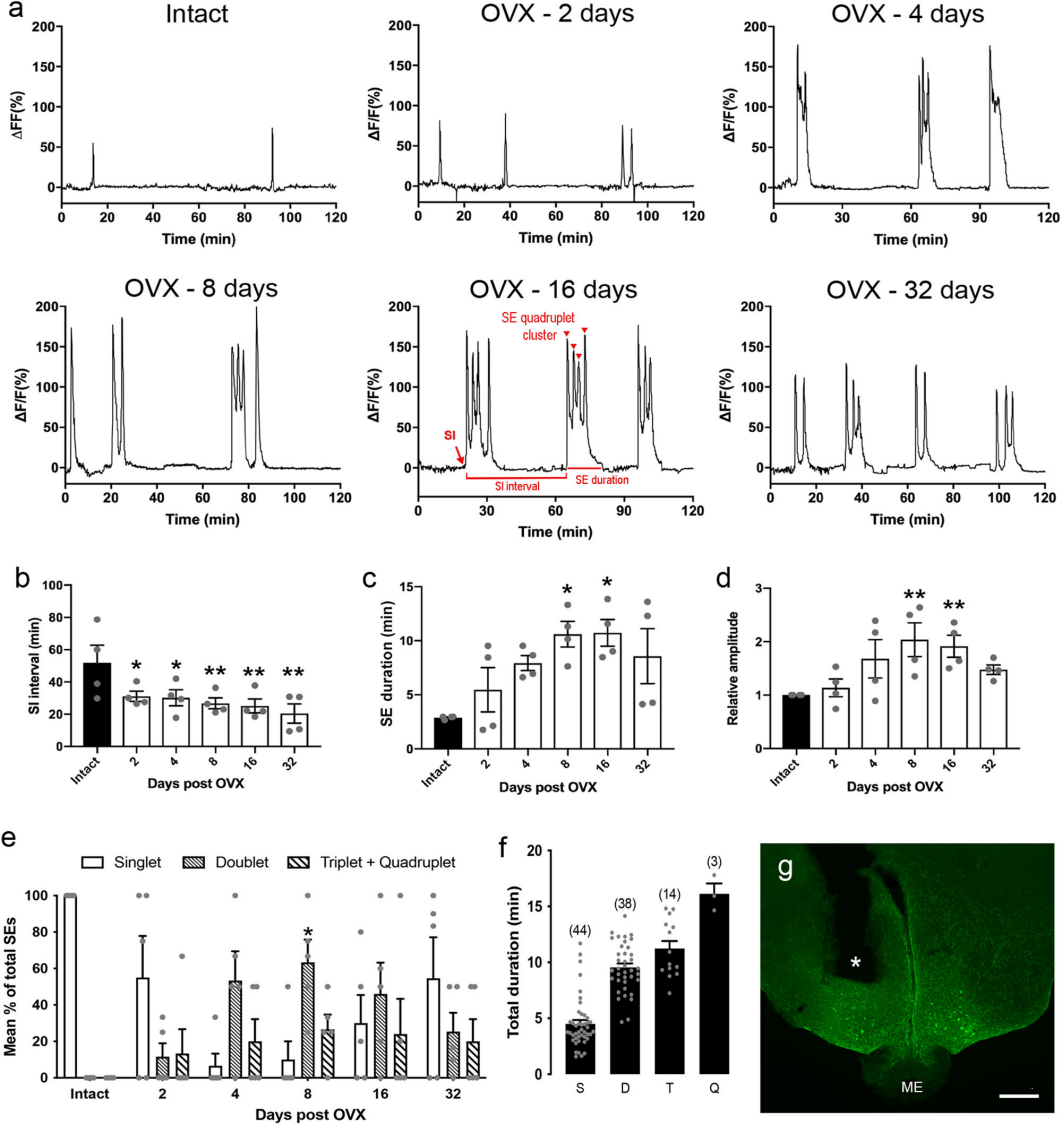
Changes in GnRH pulse generator activity with time following ovariectomy (OVX). **a** Representative example of GCaMP6 photometry recordings from ARN^KISS^ neurons of a single mouse before and 2, 4, 8, 16, and 32 days following ovariectomy. Red text on 16 day profile illustrates the parameters measured; the synchronization initiation (SI), duration and interval and the individual synchronization events (SEs) occurring within clusters (red arrowheads). **b** Graph showing the mean ± SEM and individual data points for the inter-SI interval before (Intact) and following OVX (RM one-way ANOVA; *P* = 0.006; *N* = 4) * and ** significantly different from the intact group, Dunnett’s multiple comparisons (*P* < 0.05 and 0.001). **c** Gradual increase (mean ± SEM) in the duration of SE’s following OVX (RM one-way ANOVA; *P* = 0.028; *N* = 4) * significantly different from the intact group; Dunnett’s multiple comparisons (*P* < 0.05). **d** Gradual increase (mean ± SEM) in relative SE amplitude following OVX (repeated measures one-way ANOVA; *P* = 0.004; *N* = 4) ** significantly different from the intact group; Dunnett’s multiple comparisons (*P* < 0.001). **e** Graph showing the mean (+ SEM*)* percentage of all SE’s occurring as singlets, doublets, and triplets plus quadruplets following OVX, * significantly different from the OVX day 2 group (*P* = 0.0127, Repeated measures ANOVA; *N* = 4). **f** Total duration (mean ± SEM) of SEs found in OVX mice when comprised of singlet (S), doublet (D), triplet (T), or quadruplet (Q) events. Numbers in brackets indicate the total number of SEs measured from 4 independent mice. **g** low-power photomicrograph showing the location of an optic fiber (asterisk) in relation to GCaMP-expressing kisspeptin neurons in the caudal ARN. ME median eminence. Scale bar = 200 μm. Source data are provided as a Source Data file.

Approximately 70% of ARN^KISS^ neurons express GCaMP6 and essentially all (96%) GCaMP-expressing cells are kisspeptin neurons in this AAV-injected Kiss-Cre,GCaMP6s mouse model^6^. As reported previously^24^, ARN^KISS^ neurons in intact diestrous mice exhibit abrupt transient increases in GCaMP6 fluorescence signal, termed synchronization events (SEs), approximately once every 50 min (Fig. 1b). All optic fibers recording SEs were located immediately above the ARN (Fig. 1g).

Following ovariectomy, the profile of GnRH pulse generator activity changed to one of higher frequency, longer duration, and higher amplitude SEs (*N* = 4–5; Fig. 1). Two days after ovariectomy, SEs began to be comprised of clusters of tightly coupled individual synchronizations following each synchronization initiation (SI); these occurred together as doublet, triplet and occasionally quadruplet events (Fig. 1a, e). The total duration of these events varied from 4.5 ± 0.4 min for a single SE to 16.1 ± 0.9 min for a quadruplet SE cluster (Fig. 1f) with the mean SE cluster duration increasing significantly from day 8 after ovariectomy (repeated measures (RM) one-way ANOVA, F_(5,15)_ = 3.45, *P* = 0.028; *P* < 0.01 Dunnett’s multiple comparisons) (Fig. 1c). The interval between individual synchronizations occurring within a cluster remained constant at ~4 min from post-OVX day 2 to 32 (*P* = 0.8026; one-way ANOVA F_(4,14)_ = 0.40). The percentage of SEs comprised of singlet, doublet and triplet/quadruplet events did not change markedly from day 2 to 32 with the only significant change being the proportion of doublets that increased from day 2 to Day 8 (*P* = 0.0127; RM one-way ANOVA F_(3,12)_ = 5.5, *P* = 0.025) (Fig. 1e). In addition to the change in SE profile, the frequency of SEs increased following ovariectomy with the SI interval being significantly reduced from day 2 onwards declining from 51.9 ± 10.9 to 20.4 ± 4.5 min on day 32 (RM ANOVA F_(5,15)_ = 5.20, *P* = 0.006; Dunnett’s multiple comparisons *P* = 0.002–0.024) (Fig. 1b). Finally, relative SE amplitude increased being significantly different at day 8 (*P* = 0.003) and 16 (*P* = 0.009) compared with intact values from each mouse (RM ANOVA F_(5,15)_ = 5.55, *P* = 0.004) (Fig. 1d). These recordings show that the removal of gonadal steroids results in the GnRH pulse generator operating more frequently and with prolonged, high amplitude SE clusters.

**Fig. 2:**
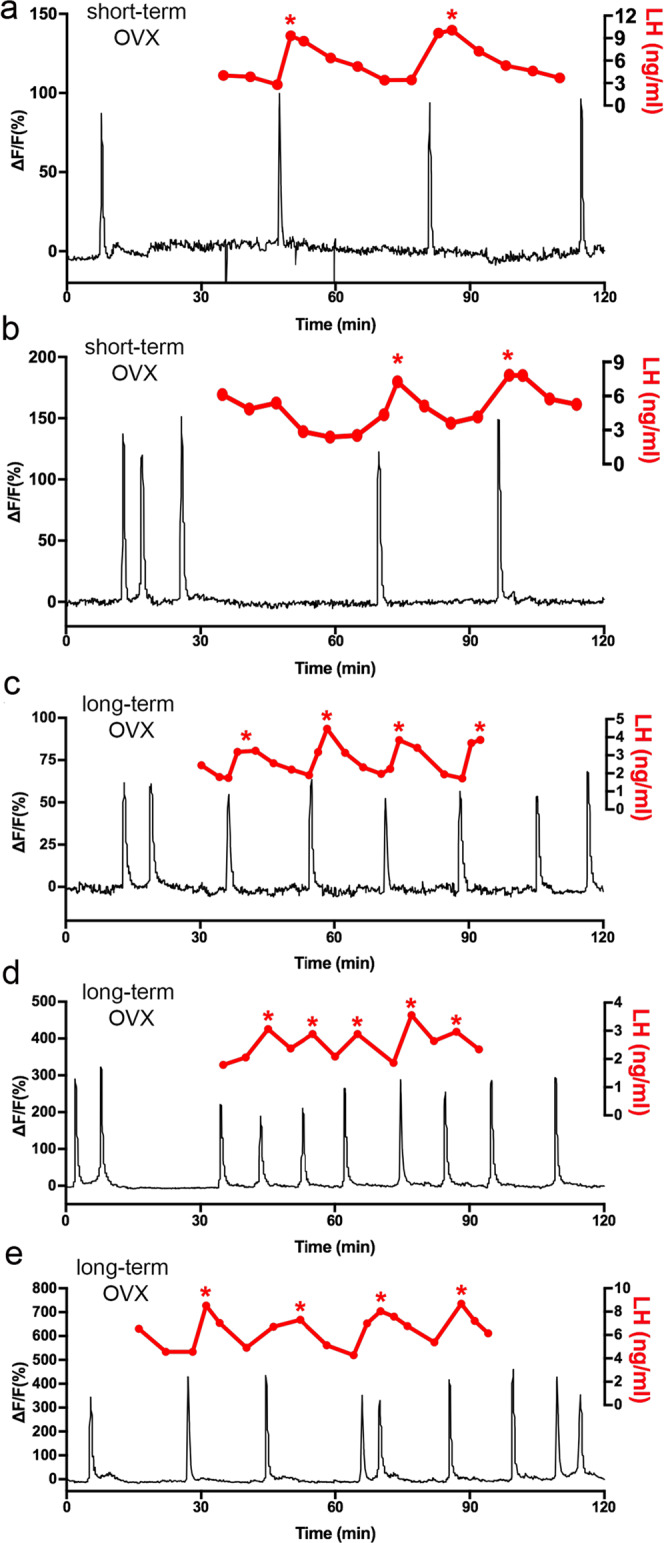
Relationship of pulse generator activity to pulsatile LH secretion in OVX mice. Representative examples from 2 short-term (~2 weeks) OVX (**a**, **b**) and three long-term (>4 weeks) OVX (**c**–**e**) mice showing the correlation between SEs recorded using GCaMP6 photometry and LH pulses measured from tail-tip blood samples. Individual LH pulses are indicated by asterisks.

To examine the relationship of these ARN^KISS^ neuron SEs to pulsatile LH, repeated 5 min tail-tip bleeding was undertaken in mice ~2 weeks and >4 weeks after ovariectomy. In the 2-week OVX mice (*N* = 4) a perfect relationship was found between SIs and LH pulses (all SIs were followed by an LH pulse and no LH pulses occurred without a preceding SI, Fig. 2a, b). In longer-term OVX mice (*N* = 4), the same relationship was found (Fig. 2c–e) although the 5 min pulse bleeding interval was insufficient when SIs occurred with an interval of 10 min or less as this only allowed the resolution of a single-point sawtooth pattern of LH secretion (Fig. 2d). Despite extensive habituation, the bleeding procedure almost always stopped the occurrence of SE clusters so that only singlet SEs occurred during the pulse bleeding. The exception was a sole doublet SE cluster that was found to be associated with a single LH pulse following the first synchronization in the doublet cluster (Fig. 2e).

### Estradiol returns arcuate kisspeptin neuron pulse generator activity to intact levels

**Fig. 3:**
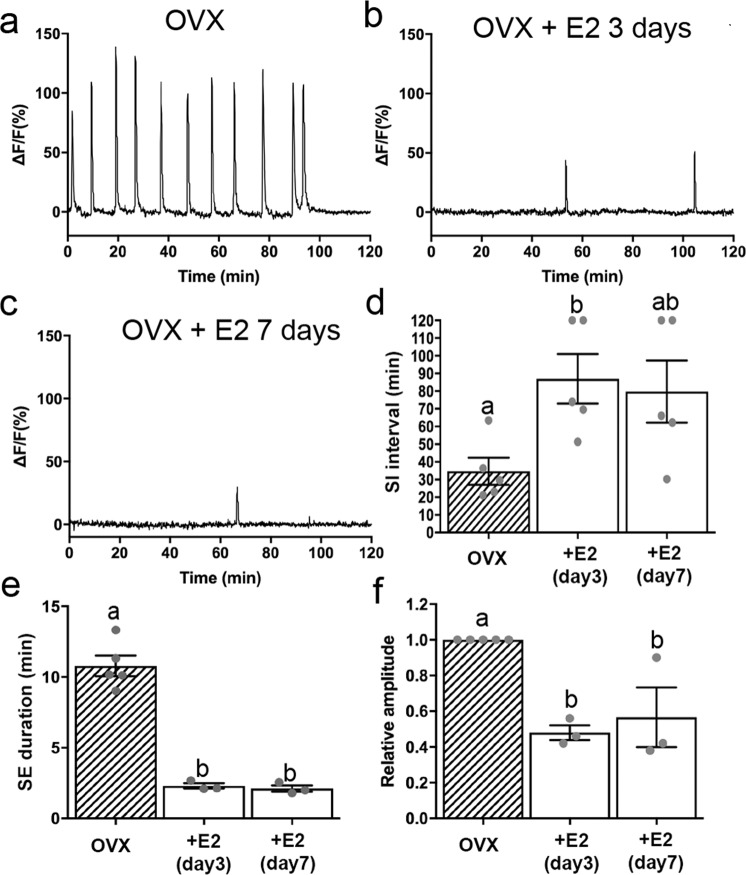
Effect of estradiol on pulse generator activity in long-term OVX mice. **a**–**c** Representative example of GCaMP6 photometry recordings from an OVX mice prior to and 3 and 7 days following treatment with estradiol. **d** Graph showing the mean ± SEM and individual data points for SI interval following estradiol replacement (one-way ANOVA; *P* = 0.040; *N* = 5). **e** Mean (±SEM) reduction in SE duration following estradiol replacement (one-way ANOVA; *P* < 0.0001; *N* = 5). **f** Mean (±SEM) decrease in relative SE amplitude following estradiol replacement (one-way ANOVA; *P* = 0.0023; *N* = 5). Significant differences are indicated by bars with different letter, Dunnett’s multiple comparisons (*P* < 0.01 or 0.001 in all cases). Source data are provided as a Source Data file.

To assess the role of 17-β−estradiol (E2) in regulating ARN^KISS^ neuron SEs, OVX mice were given a 4 μg/mouse s.c. Silastic capsule E2 replacement regimen recently described to accurately model estrogen negative feedback^25^. This was found to return the activity of the pulse generator to an intact-like state by 3 days with the effects persisting at day 7 (Fig. 3a–c). Estradiol replacement significantly increased SI interval (*P* = 0.040; one-way ANOVA F_(2,12)_ = 4.3; Fig. 3d) and reduced SE duration (*P* < 0.0001; one-way ANOVA F_(2,8)_ = 71.64; Fig. 3e). There was a complete loss of SE cluster events with a return to singlet SEs. In addition, the amplitude of SEs was significantly reduced (*P* = 0.0023; one-way ANOVA F_(2,8)_ = 14.19; Fig. 3e). The SI interval (79 ± 18 min) and SE duration (2.1 ± 0.3 min) of 7-day E2 mice were not significantly different to that of intact diestrous mice (52 ± 11 min, 2.9 ± 0.1 min; all *P* > 0.05, Mann–Whitney tests, Fig. 1). The amplitude cannot be compared directly but is approximately doubled by OVX (Fig. 1d) and then halved by E2 treatment (Fig. 3f). These data indicate that estradiol is one of the principal gonadal steroids suppressing pulse generator activity in intact mice.

### Arcuate kisspeptin neuron pulse generator activity in Kiss1 neuron-specific ESR1 KO mice

**Fig. 4:**
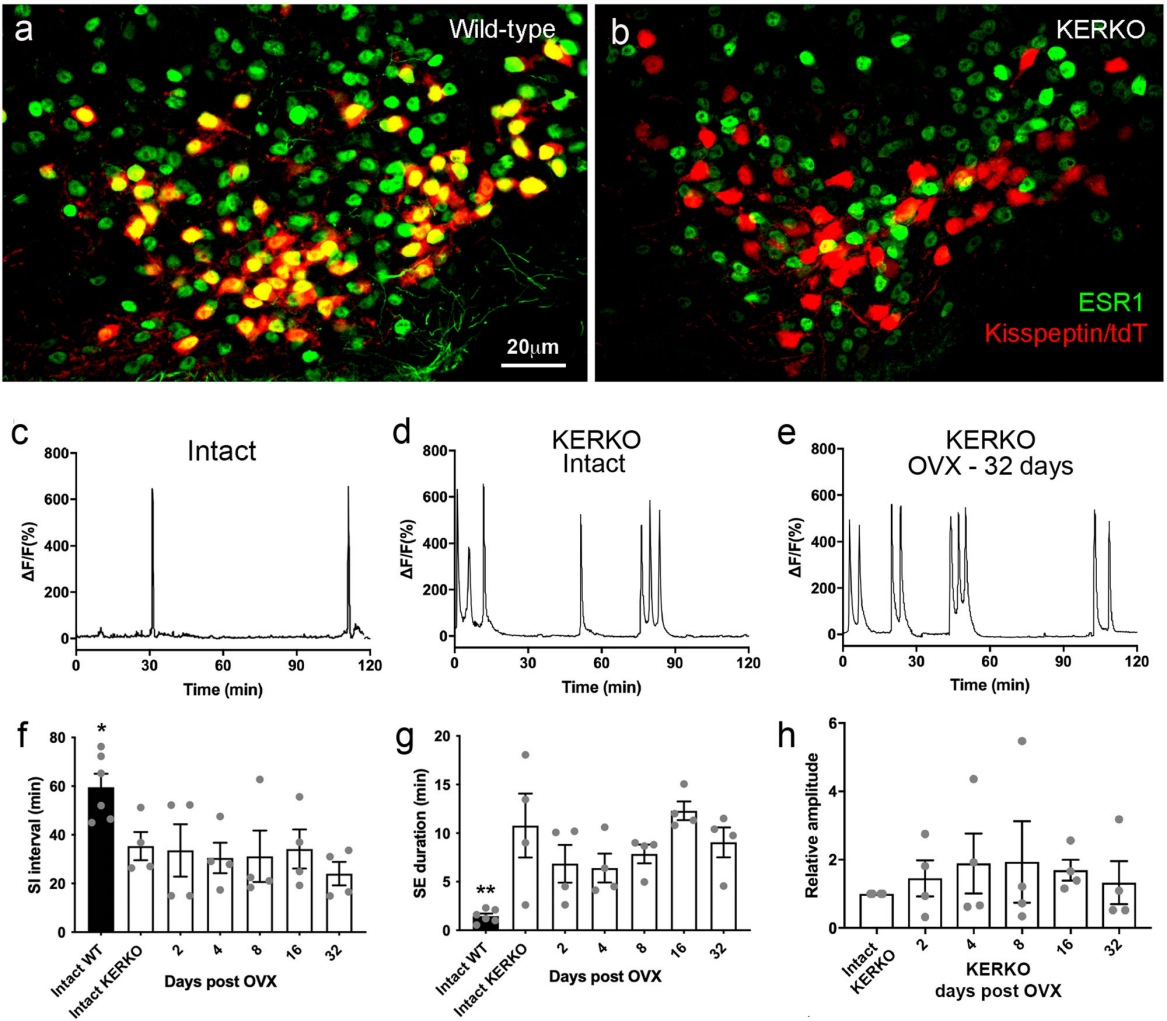
GnRH pulse generator activity in Kiss1-specific ESR1 knockout (KERKO) mice. **a**, **b** Representative examples of dual-label immunofluorescence for tdT (kisspeptin) and ESR1 (green) in wild-type (**a**, *N* = 4) and KERKO (**b**, *N* = 5)) mice. Almost all tdT neurons co-express ESR1 (yellow nuclei) while none occur in the KERKO mouse. **c** Representative example of GCaMP6 photometry recordings from an intact mouse wild-type at the *Esr1* locus. **d**, **e** photometry recordings from KERKO mice when intact and 32 days after OVX. **f**–**h** Graphs showing mean ± SEM and individual data points for photometry parameters recorded from intact WT mice and KERKO mice before (intact) and 2, 4, 8, 16, and 32 days following OVX. Intact WT versus Intact KERKO *P = 0.038 and **P = 0.010 (Mann–Whitney test; *N* = 4)). No parameters changed at any time point after OVX (*P* > 0.05, one-way ANOVA, see text for exact values). Source data are provided as a Source Data file.

To test the hypothesis that estrogen negative feedback occurs through ESR1 expressed by the ARN^KISS^ neurons, we used GCaMP fiber photometry to examine SEs in kisspeptin cell-specific *Esr1* knockout (KERKO) mice. Dual-label immunofluorescence showed that 96 ± 2% of ARN tdT cells (representing ARN Kiss1-Cre cells) expressed ESR1 in wild-type mice (*N* = 5) while KERKO^−/−^ mice had a complete absence of ESR1 immunoreactivity in ARN^KISS^ neurons (*N* = 4) (Fig. 4a, b).

Fiber photometry recordings demonstrated that AAV-injected intact female Kiss-Cre,GCaMP6s,KERKO^−/−^ mice (*N* = 4) exhibited a pattern of ARN^KISS^ neuron activity comprised of frequent clusters of SEs that was significantly different to that of intact control Kiss-Cre,GCaMP6s,KERKO^+/+^ mice (*N* = 6) (Fig. 4C) but the same as OVX mice recorded previously (Fig. 1). The SI interval was significantly decreased (*P* = 0.038, Mann–Whitney test) and SE duration increased (*P* = 0.01, Mann–Whitney test) in intact KERKO mice compared to intact wild-type diestrous mice (Fig. 4 c, d, f, g). Comparing the ARN^KISS^ neuron activity of KERKO^−/−^ mice with 32-day OVX mice wild-type at the *Esr1* locus (Fig. 1b–d) revealed no significant differences for SI interval (*P* = 0.343, Mann–Whitney) or SE duration (*P* = 0.886, Mann–Whitney).

These observations raise the possibility that the effects of estradiol on the synchronization behavior of ARN^KISS^ neurons are mediated entirely by ESR1 expressed by these cells. To examine whether estradiol actions at other cells may have additional roles, Kiss-Cre,GCaMP6s,KERKO^−/−^ mice (*N* = 4) were ovariectomized to remove estradiol actions outside the kisspeptin neurons and fiber photometry used to examine ARN^KISS^ neuron synchronization behavior from day 2 to day 32 (Fig. 4d). No changes were observed for any parameter at any time point following ovariectomy (*P* > 0.05, one-way ANOVA F_(6,22)_ = 0.3209-2.48; Fig. 4f–h).

While supporting the notion that no other cell type contributes to the negative feedback actions of E2 on ARN^KISS^ synchronizations, ovariectomy results in the removal of all gonadal factors from circulation. To address the role of estradiol specifically, the synchronization activity of the four OVX Kiss-Cre,GCaMP6s,KERKO^−/−^ mice was assessed immediately before and then 3 and 7 days after being given the E2 negative feedback replacement regimen described above. This had no effect on any parameter with the SI interval (*P* = 0.790; one-way ANOVA F_(2,6)_ = 0.25), SE duration (*P* = 0.160; one-way ANOVA F_(2,6)_ = 0.25), and relative amplitude (*P* = 0.601; one-way ANOVA F_(2,6)_ = 0.55) all unchanged by E2 treatment (Table 1). Together, these observations indicate that ESR1 within ARN^KISS^ neurons accounts for all the suppressive actions of E2 on the GnRH pulse generator.

**Table 1:** ARN^KISS^ neuron synchronization event (SE) dynamics in OVX KERKO mice with and without E2 treatment

	SI frequency /60 min	SI interval (min)	SE frequency/60 min	Intra-cluster SE intervals (min)	SE duration (min)	Relative amplitude
OVX	2.5 ± 0.29	22.0 ± 6.0	4.5 ± 0.29	4.6 ± 0.4	11.0 ± 0.51	1
OVX + E2 day 3	2.3 ± 0.33	18.0 ± 2.6	3.5 ± 0.29	4.3 ± 0.3	9.1 ± 0.53	1.2 ± 0.33
OVX + E2 day 7	3.0 ± 0.29	20.0 ± 2.0	4.7 ± 0.44	4.7 ± 0.3	9.7 ± 0.27	1.5 ± 0.44

### CRISPR knockdown of ESR1 in adult ARN^KISS^ neurons

While powerful, there are caveats to using KERKO mice to understand the mechanism of estrogen negative feedback; normal pathways of estradiol action are very likely altered during development following *Esr1* deletion^26,27^ and, in addition, *Esr1* is deleted in all kisspeptin-expressing cells in the body. To overcome these issues, we employed a CRISPR gene editing approach^28^ to knockdown ESR1 selectively in ARN^KISS^ neurons in the adult female mouse.

### Design of gRNA, testing and in vitro validation of gRNAs

Six guide RNAs were designed to target different domains of *Esr1* (NM_007956) in both the sense and antisense orientation (Supplementary Fig. 1a, b). To test gRNA efficacy in vitro, the ESR1-expressing hypothalamic cell line mHypo-CLU189-A was genetically modified to stably express Cas9 (CLU189-Cas922C) and transduced with AAV-U6-gRNA-EGFP for all six gRNA. gRNA-1, −2, −3, and −6 were found to be the most effective in reducing *Esr1* mRNA levels by 20–30% (Supplementary Fig. 1c). gRNA-3 and −6, representing a sense and antisense combination targeting different domains, were chosen for further work. To allow operation with the Cas9/EGFP mouse in vivo, the U6 promoter, gRNA, and scaffold cassette of the PX552 construct was PCR amplifed and subcloned into pAAV-Ef1a-mCherry (AddGene #114470) to generate AAV1-U6-gRNA3/6-Ef1a-mCherry. CLU189-Cas922C cells transduced with AAV1-U6-gRNA3-Ef1a-mCherry or AAV1-U6-gRNA6-Ef1a-mCherry exhibited significant 50% or 30% decreases *Esr1* mRNA levels (Supplementary Fig. 1d).

### Effects of gRNAs on ESR1 expression in vivo

To assess the effects of gRNA-3 and −6 in vivo, initial experiments were undertaken in *Vgat-Cre,LSL-Cas9-EGFP* mice given unilateral injections AAV1-U6-gRNA(3 or 6)-Ef1α-mCherry into the medial preoptic area. Three weeks later this was found to have resulted in 72 ± 5% and 78 ± 3% reductions, respectively, in the numbers of ESR1-immunoreactive EGFP-expressing neurons on the injected side of the brain compared with the non-injected side using gRNA-3 (*N* = 4) and gRNA-6 (*N* = 4)(Supplementary Fig. 2).

**Fig. 5:**
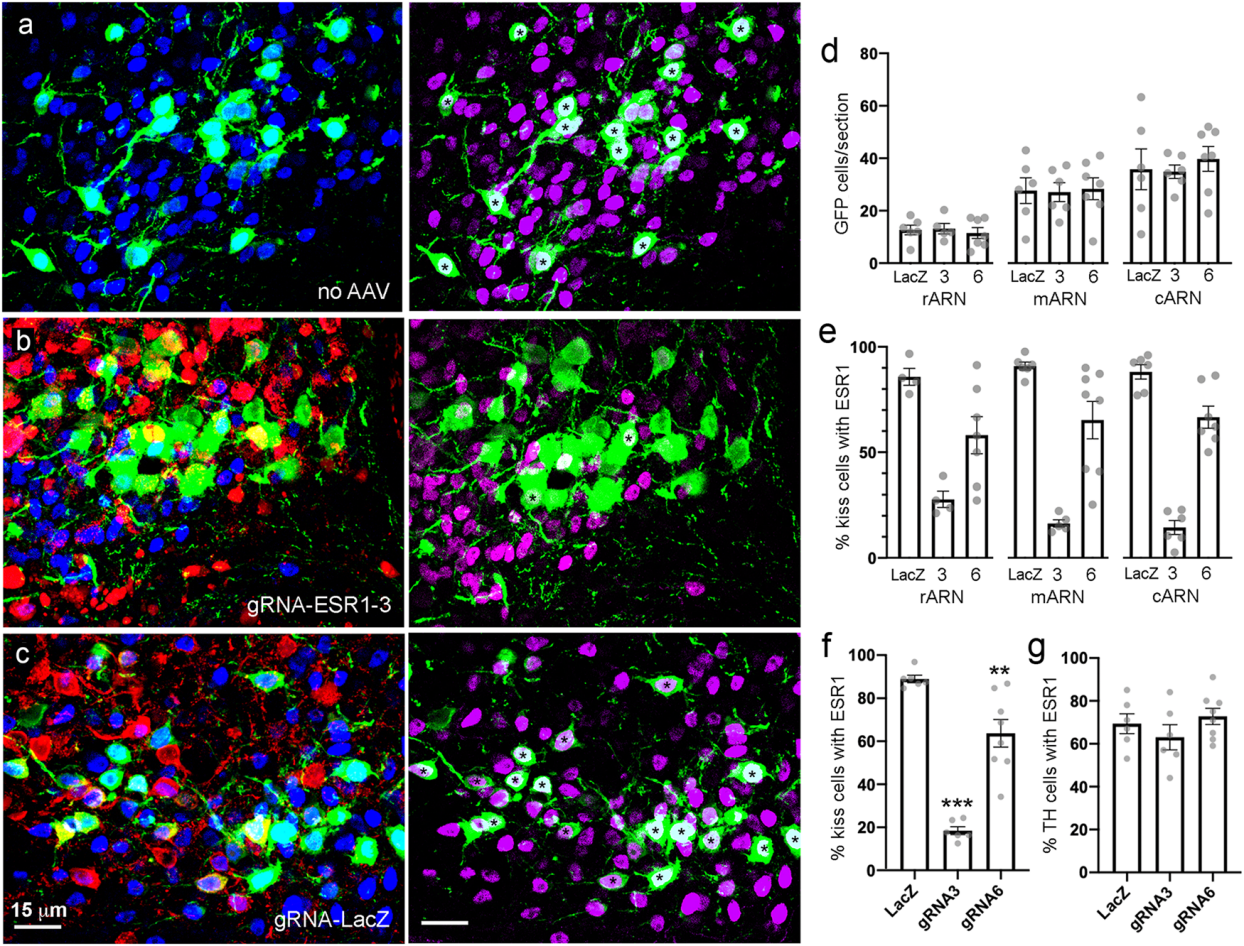
CRISPR-Cas9 knockdown of ESR1 expression in adult ARN^KISS^ neurons. **a**–**c** Photomicrographs showing EGFP/Cas9 (green), ESR1 (blue), and mCherry (red) expression in the ARN of mice receiving no gRNA injection (**a**), gRNA-ESR1-3 (**b**), and gRNA-LacZ (**c**). Almost all GFP cells co-express ESR1 (blue/light blue nuclei) in **a** and **c** compared with only two cells in **b**. **a** and **b** are from opposite sides of the ventricle of mouse 16265 that received a unilateral injection of gRNA-ESR1-3. To facilitate identification of dual-labeled cells, the same photomicrographs are shown to the right with the mCherry channel removed and ESR1 now displayed in magenta. Dual-labeled cells have an asterisk in the nucleus. **d** Mean ± SEM and individual data points showing numbers of EGFP/Cas9 cells detected per section in the presence of gRNA-LacZ, gRNA-ESR1-3 (3) and gRNA-ESR1-6 (6) in the rostral (rARN), middle (mARN) and caudal (cARN) (*N* = 6 or 7 per experimental group). **e** Mean ± SEM and individual data points showing % of kisspeptin (EGFP/Cas9) cells expressing ESR1 in the presence of gRNA-LacZ, gRNA-ESR1-3, and gRNA-ESR1-6 at the three rostro-caudal ARN levels (*N* = 4–7 per experimental group). **f** Mean ± SEM and individual data points for all regions of the ARN combined showing % of kisspeptin (EGFP/Cas9) cells expressing ESR1 in the presence of gRNA-LacZ, gRNA-ESR1-3 (gRNA3), and gRNA-ESR1-6 (gRNA6). ***P < 0.0001, **P = 0.002, post hoc Dunnett’s test versus LacZ (*N* = 6 or 7 per experimental group). **g** Mean ± SEM and individual data points showing % of tyrosine hydroxylase (TH) cells expressing ESR1 in the presence of gRNA-LacZ, gRNA-ESR1-3 (gRNA3), and gRNA-ESR1-6 (gRNA6) (*N* = 6 or 8 per experimental group). Source data are provided as a Source Data file.

The effects of the same gRNAs on ESR1 expression in ARN^KISS^ neurons was evaluated in *Kiss1-Cre,LSL-Cas9-EGFP* mice at the end of the in vivo series of studies (Fig. 5). Cells expressing EGFP/Cas9 were only detected in the ARN and exhibited the known rostro-caudal distribution of ARN^KISS^ neurons with the numbers increasing from the rostral ARN through to the caudal ARN (Fig. 5d). The numbers of EGFP cells were not different in mice receiving gRNA-LacZ, gRNA-3, or gRNA-6 (Fig. 5d). Given the use of bilateral stereotaxic injections in an elongated nucleus such as the ARN, there was variable gRNA distribution and consequent ESR1 knockdown amongst different mice, including some with only unilateral injections (Supplementary Fig. 3). For the control LacZ gRNA (AAV1-U6-gRNA-LacZ-Ef1α-mCherry) injections, 86 ± 4%, 91 ± 2%, and 88 ± 3% of kisspeptin neurons located in the rostral, middle and caudal ARN injected with gRNA expressed ESR1, respectively (*N* = 4–6 per ARN subdivision, Fig. 5c, e). Together, 89 ± 2% of ARN^KISS^ neurons expressed ESR1 in the presence of gRNA-LacZ (Fig. 5f). In three mice with unilateral gRNA-LacZ injections at one or more levels of the ARN, 92 ± 3% of kisspeptin neurons expressed ESR1 in the presence of the gRNA compared with 95 ± 3% on the opposite non-injected side.

Mice receiving AAV1-U6-gRNA-3-Ef1α-mCherry (*N* = 6) had bilateral and unilateral gRNA injections (Fig. 5a, b; Supplementary Fig. 2) with 28 ± 4%, 16 ± 2%, and 15 ± 3% of ARN^KISS^ neurons at the rostral, middle and caudal levels expressing ESR1 in the presence of gRNA-3 (Fig. 5b, e). Overall, gRNA-3 resulted in an 80% reduction in ESR1 expression by ARN^KISS^ neurons (*P* < 0.0001; one-way ANOVA F_(2,17)_ = 52.37, post hoc Dunnett’s test versus LacZ *P* < 0.0001; Fig. 5f). Mice receiving AAV1-U6-gRNA-6-Ef1α-mCherry (*N* = 8) also had bilateral and unilateral gRNA injections (Supplementary Fig. 3). In this case, 58 ± 9%, 65 ± 9%, and 67 ± 5% of kisspeptin neurons expressed ESR1 in the presence of gRNA-6 in the rostral, middle, and caudal ARN, respectively (Fig. 5e). This represented an overall small but significant 28% reduction in ESR1 expression by ARN^KISS^ neurons (*P* < 0.0001; one-way ANOVA F_(2,17)_ = 52.37, post hoc Dunnett’s test versus LacZ *P* = 0.002; Fig. 5f).

To examine the selectivity of CRISPR gene editing in kisspeptin neurons, the numbers of dopaminergic neurons in the ARN expressing ESR1 were quantified with dual-label immunohistochemistry at the level of the middle ARN (Supplementary Fig. 4). Approximately 25–30 tyrosine hydroxylase (TH)-immunoreactive neurons were detected per section in all mice with no differences in ESR1 expression; 69 ± 5%, 63 ± 6%, and 72 ± 4% of TH neurons expressed ESR1 in the presence of gRNA-LacZ (*N* = 6), gRNA-3 (*N* = 6), and gRNA-6 (*N* = 8) (*P* = 0.3462; one-way ANOVA F_(2,17)_ = 1.13; Fig. 5g, Supplementary Fig. 4).

Together, these data demonstrate that the two ESR1 gRNAs result in the selective suppression of ESR1 in adult ARN^KISS^ neurons although gRNA-3 drives an 80% knockdown compared to a more modest ~30% decrease with gRNA-6.

### Effects of CRISPR knockdown of ESR1 in adult ARN^KISS^ neurons on LH pulsatility and cyclicity

The estrous cycles of *Kiss1-Cre,LSL-Cas9* mice were determined over a 3-week period before stereotaxic injection of AAV-gRNA into the ARN and then again for 3 weeks after at least a 3-week post-surgical interval. Mice exhibited normal estrous cycles with an average length of ~5 day before and after gRNA injections regardless of whether they received unilateral or bilateral injections of gRNA-LacZ (*N* = 6), gRNA-3 (*N* = 6), or gRNA-6 (*N* = 8) (Supplementary Fig. 3). The estrous cycles of individual mice with the largest global gRNA-3 and gRNA-6 knockdowns of ESR1 in ARN^KISS^ neurons (Supplementary Fig. 3) are shown in Supplementary Fig. 5.

**Fig. 6:**
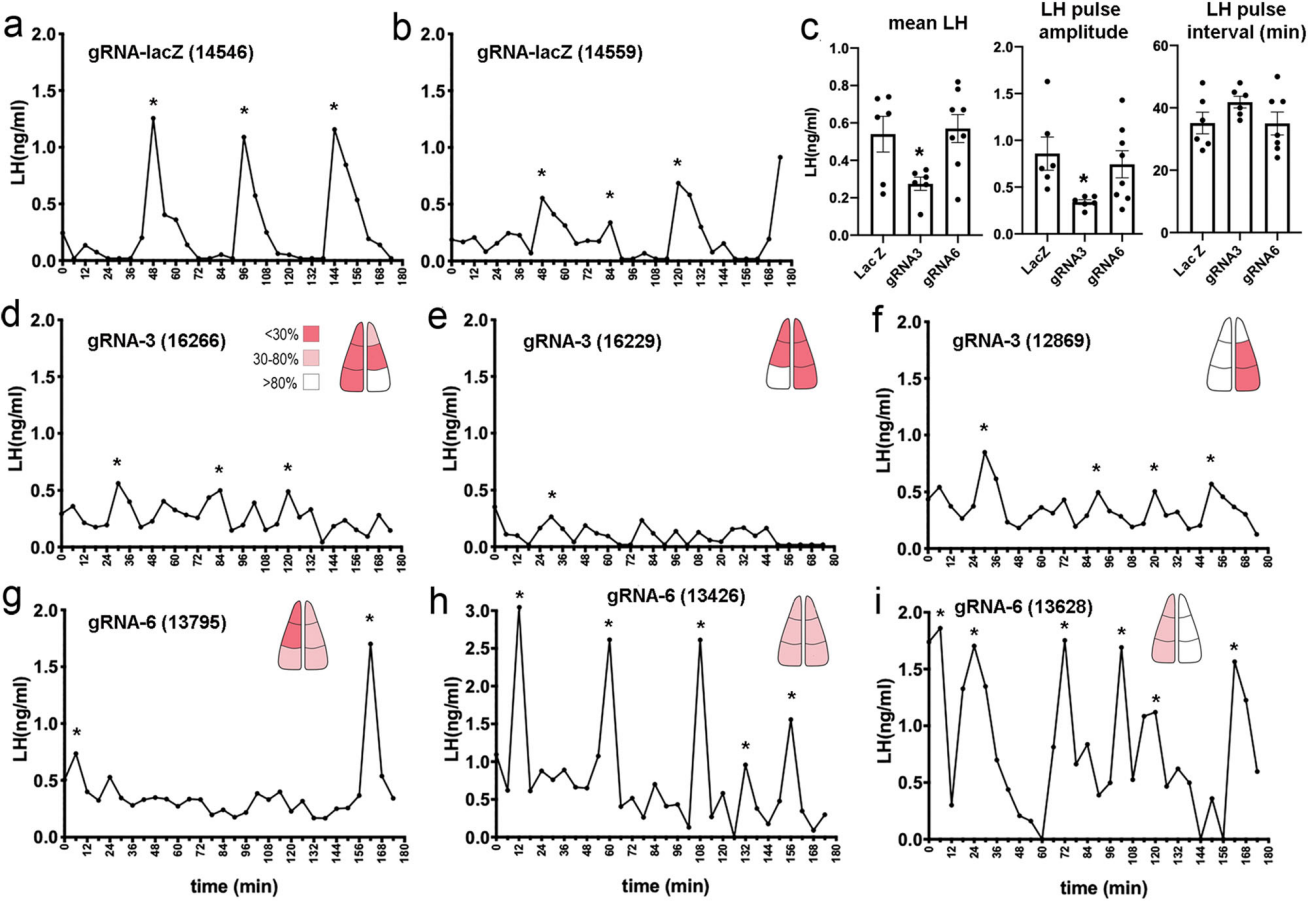
Effects on pulsatile LH secretion of CRISPR-Cas9 knockdown of ESR1 expression in adult ARN^KISS^ neurons. **a**, **b** Examples of LH pulse profiles in *Kiss1-Cre,LSL-Cas9-EGFP* mice given gRNA-LacZ. Numbers in brackets refer to animal number. Asterisks indicate LH pulses. **c** Mean ± SEM LH levels, pulse amplitudes and pulse intervals in the experimental groups receiving gRNA-LacZ (LacZ, *N* = 6), gRNA-ESR1-3 (gRNA3, *N* = 6), and gRNA-ESR1-6 (gRNA6, *N* = 8). *P ≤ 0.05, post hoc Dunnett’s test versus LacZ. A pulse interval of 156 min (see G.) is not shown for gRNA6. **d-f**, Examples of LH pulse profiles in *Kiss1-Cre,LSL-Cas9-EGFP* mice given gRNA-ESR1-3. Schematics give a flat-map bilateral rostro-caudal representation of the distribution and extent of ESR1 knockdown in kisspeptin neurons; red <30% Kiss with ESR1, pink 30–80%, white >80%. **g**–**i** Examples of LH pulse profiles in *Kiss1-Cre,LSL-Cas9-EGFP* mice given gRNA-ESR1-6. Asterisks indicate LH pulses detected with PULSAR. Source data are provided as a Source Data file.

Following estrous cycle monitoring, pulsatile LH secretion was evaluated in diestrus using 5 min interval tail-tip bleeding for 180 min^29,30^ and analyzed using PULSAR-Otago^25^. Mice receiving gRNA-LacZ (*N* = 6) exhibited typical LH pulses for intact diestrous mice with an interval of 35.2 ± 3.5 min, amplitude of 0.86 ± 0.18 ng/mL and a mean LH level of 0.54 ± 0.10 ng/mL (Fig. 6a, b). Mice receiving gRNA-ESR1-3 (*N* = 6) exhibited an unusual, low-level pattern of fluctuating LH secretion with many small increases in LH that were not considered pulses using the PULSAR criteria prescribed for intact female mice^25^ (Fig. 6d–f). This was seen in all mice regardless of whether they had received bilateral or unilateral ESR1 knockdown but was most prominent in those receiving bilateral gRNA (Fig. 6d–f). As the suppression of ESR1 enhances activity, it is most likely that the elevated pulse generator activity occurring unilaterally in mice with unilateral ESR1 knockdown was sufficient to drive abnormal GnRH secretion. Although pulses were under-estimated, PULSAR-detected LH pulses had a significantly reduced pulse amplitude (0.34 ± 0.26 ng/mL; *P* = 0.0376, one-way ANOVA F_(2,17)_ = 3.60, post hoc Dunnett’s test versus LacZ *P* = 0.039; Fig. 6c) and mean LH levels were also significantly reduced (0.28 ± 0.04 ng/mL; *P* = 0.0249, one-way ANOVA F_(2,17)_ = 4.63, post hoc Dunnett’s test versus LacZ *P* = 0.050; Fig. 6c). Mice receiving gRNA-ESR1-6 (*N* = 8) exhibited a range of pulsatile LH patterns extending from low-level fluctuations (Fig. 6g) to relatively normal intact LH pulses (Fig. 6h, i). As a group, mean LH (0.57 ± 0.10 ng/mL), pulse amplitude (0.74 ± 0.15 ng/mL) and pulse interval (35.0 ± 3.7 min) were not different to that of gRNA-LacZ mice (*P* > 0.05, post hoc Dunnett’s test versus LacZ) (Fig. 6c). A modest but significant correlation (*P* = 0.008, r = 0.5773, Spearman Correlation) between the degree of overall ESR1 knockdown in ARN^KISS^ neurons and the amplitude of PULSAR-detected LH pulses was found amongst all mice in the three experimental groups (Supplementary Fig. 6).

**Fig. 7:**
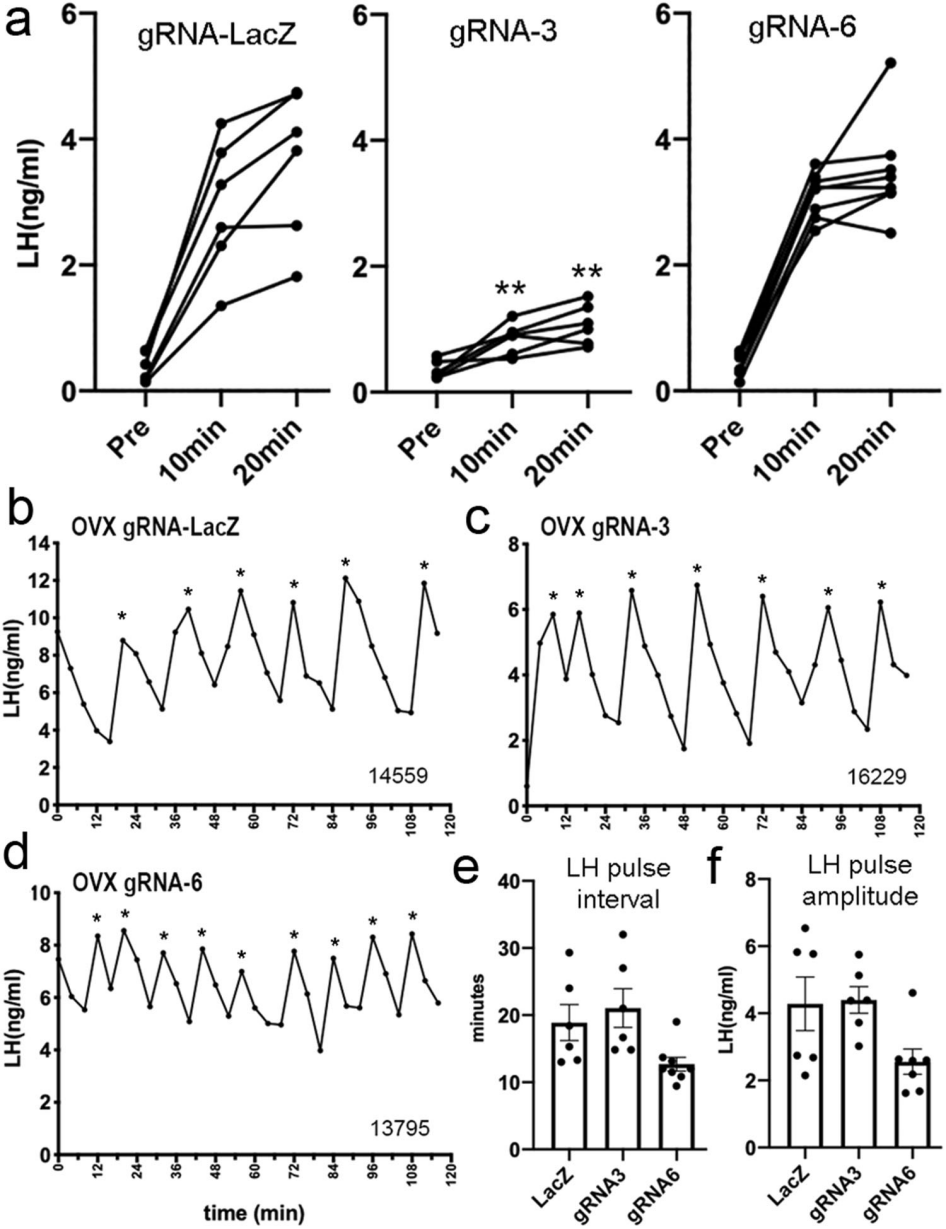
Pituitary function and effects of ovariectomy in mice with knockdown of ESR1 in adult ARN^KISS^ neurons. **a** Individual profiles of LH secretion in mice from the three gRNA groups showing LH levels before (Pre) and then 10 and 20 min following a bolus s.c. administration of GnRH. **P = 0.002 at both time points versus LacZ (Mann–Whintey). **b**–**d** Representative LH pulse profiles obtained from 3-week ovariectomized mice from the three gRNA groups. Asterisks indicate pulses detected by PULSAR and numbers identify the individual mice (their intact profiles are shown in Fig. 6). **e**, **f** Mean ± SEM and individual data points for LH pulse intervals and amplitudes in 3-week OVX mice from the gRNA-LacZ (LacZ, *N* = 6), gRNA-ESR1-3 (gRNA3, *N* = 6), and gRNA-ESR1-6 (gRNA, *N* = 7) experimental groups. No significant differences were detected between groups. Source data are provided as a Source Data file.

The gRNA-ESR1-3 mice demonstrated an unexpected LH profile comprised of many small amplitude LH pulses. To explore the underlying mechanism for these reduced amplitudes, the gRNA mice were given a bolus injection of GnRH and the pituitary LH response examined 10 and 20 min later during the diestrous stage. This revealed a marked suppression in the ability of GnRH to release LH in gRNA-ESR1-3 mice compared with gRNA-LacZ mice at both time points (*P* = 0.002) (Fig. 7a). In contrast, gRNA-ESR1-6 animals displayed normal LH responses to exogenous GnRH (Fig. 7a).

These observations indicate that the ability of gonadotrophs to release LH is markedly impaired in gRNA-ESR1-3 mice. This could result from high frequency episodic GnRH drive to a pituitary that remains under direct estrogen negative feedback. To test this hypothesis, estrogen negative feedback was removed by ovariectomizing all gRNA mice and pulsatile LH secretion examined 3 weeks later with 4 min interval tail-tip pulse bleeding over 2 h. Mice exhibited typical high amplitude (~4 ng/mL pulses), frequent (~20 min interval) LH pulses following ovariectomy (Fig. 7b–d). No significant differences were detected between gRNA-LacZ and gRNA-ESR1-3 mice with ~4 ng/mL amplitude pulses occurring approximately every 20 min in both groups (Fig. 7b, c, e, f). The ovariectomized gRNA-ESR1-6 mice appeared to display more frequent, smaller amplitude LH pulses compared to gRNA-LacZ mice although this was not significantly different (post hoc Dunnett’s multiple comparisons tests versus gRNA-LacZ, *P* = 0.095 and *P* = 0.067, respectively; Fig. 7d–f). These results indicate that releasing the pituitary from estrogen negative feedback by OVX in gRNA-ESR1-3 mice enables the pituitary to follow the enhanced pulse generator activity.

### Effects of CRISPR ESR1 knockdown on adult ARN^KISS^ neuron synchronization activity

**Fig. 8:**
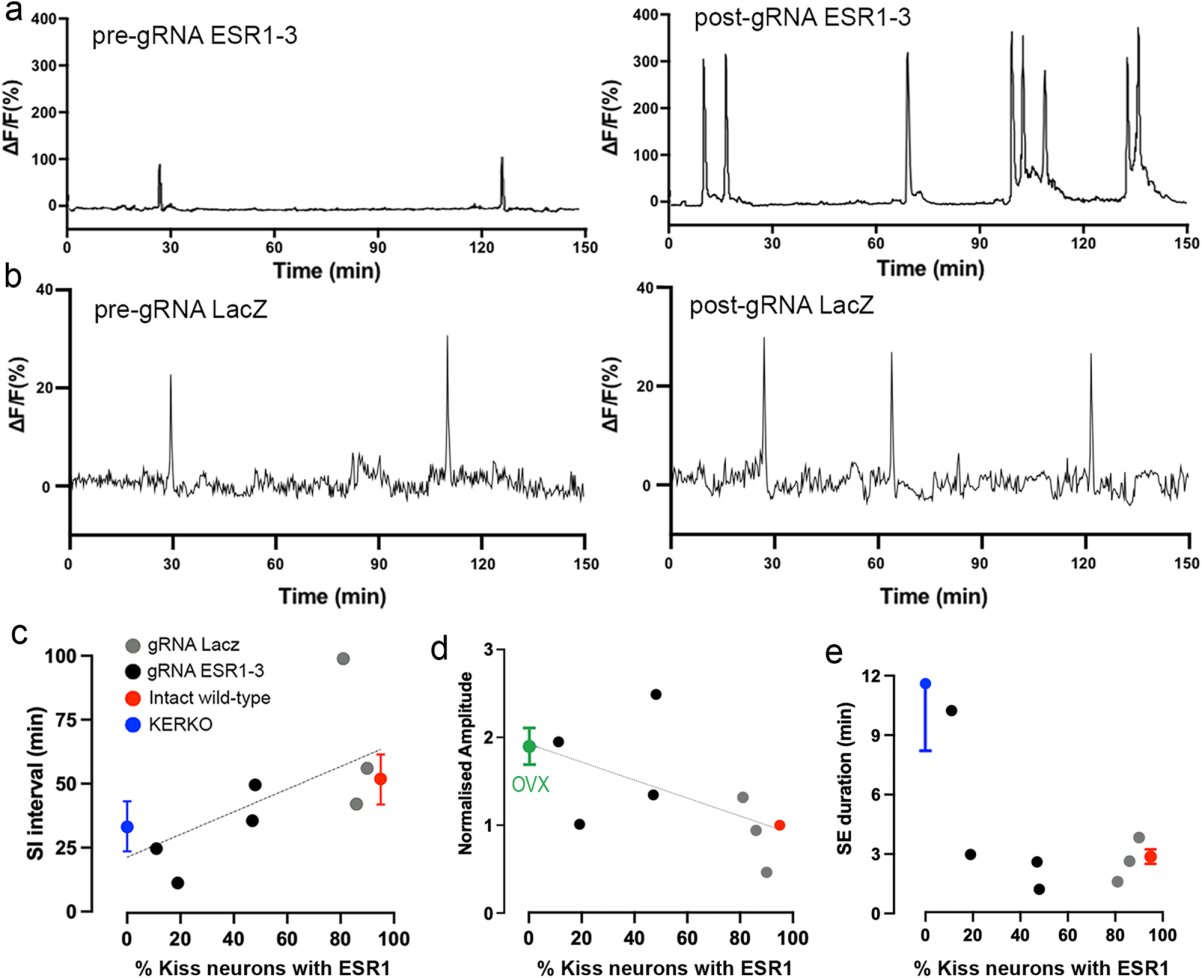
Effects of CRISPR knockdown of ESR1 on ARN^KISS^ neuron synchronization events (SEs). **a** Photometry traces before and 3 weeks after infusion of gRNA ESR1-3 into the ARN. **b** Photometry traces before and 3 weeks after infusion of control gRNA LacZ into the ARN. **c** Individual SI intervals of SEs from mice given gRNA LacZ (gray, *N* = 3), gRNA ESR1-3 (black, *N* = 4) and, for comparison, the mean ± SEM values for the same parameter recorded from intact wild-type (red) and KERKO (blue) mice plotted against the percentage of ARN^KISS^ neurons expressing ESR1. **d** Normalized SE amplitude plotted against % kisspeptin with ESR1 for individual CRISPR mice (*N* = 3 or 4 per experimental group) and, for comparison, mean ± SEM values from OVX (green) and intact mice. Values show amplitude normalized to pre-OVX or pre-AAV-gRNA infusion. **e** SE duration values for individual CRISPR mice (*N* = 3 or 4 per experimental group) and, for comparison, mean ± SEM values for KERKO and intact mice (*N* = 6 for each experimental group). Source data are provided as a Source Data file.

The above data show that the selective removal of ESR1 from adult ARN^KISS^ neurons results in a GnRH pulse generator operating at high frequency but that this activity is obscured when measuring LH secretion due to suppressed pituitary responsiveness. To evaluate the effects of acute ARN^KISS^ ESR1 CRISPR knockdown on pulse generator activity directly, we combined GCaMP6 photometry with acute CRISPR gene editing in adult mice. Mice were prepared for GCaMP fiber photometry as normal but with the exception that optical fibers carrying a micro-infusion cannula were implanted into female *Kiss1-Cre,LSL-Cas9* mice. Mice examined on diestrus before gRNA infusion exhibited typical SEs with an SI interval of 60.0 ± 9.5 min and SE duration of 2.0 ± 0.3 min (*N* = 7). Mice were divided into two groups at random with one having AAV-gRNA-LacZ (*N* = 3) and the other AAV-gRNA-ESR1-3 (*N* = 4) microinfused (1 μL) into the ARN. The effects on pulse generator SEs were examined 3–4 weeks later. Mice receiving the gRNA-LacZ showed consistent, normal pulse generator activity (Fig. 8b–e) whereas individual mice with gRNA-ESR1-3 exhibited a variety of different patterns ranging from the complete OVX profile (Fig. 8a) to relatively normal intact SEs (Fig. 8c–e). Histological assessment of ESR1 expression in ARN^KISS^ neurons demonstrated that gRNA-LacZ mice had 81–90% of ARN^KISS^ neurons expressing ESR1 whereas gRNA-ESR1-3 mice had 11–48%. These levels of ESR1 knockdown segregated with the observed pulse generator activity of each mouse (Fig. 8c–e). For both SI interval (Fig. 8c) and amplitude (normalized to the pre-gRNA conditions) (Fig. 8d), there appeared to be a linear relationship between the degree of ESR1 knockdown in kisspeptin neurons and their SE profile with the highest knockdown mice (<20% ESR1 co-expression) being very similar to KERKO mice that have complete ESR1 deletion. Mice with ~40% ESR1 co-expression exhibited parameters intermediate between those of gRNA-LacZ/intact wild-type mice and KERKO mice (Fig. 8c, d). In contrast, no linear relationship existed for SE duration with KERKO parameters only being observed in the mouse with the greatest level of ESR1 knockdown (11%) (Fig. 8e).

## Discussion

The definition of the estrogen negative feedback mechanism has received considerable attention. However, its investigation has been handicapped by technical limitations and the necessity to interpret changes in neural activity indirectly through pituitary gonadotropin secretion. The ability to record ARN^KISS^ neuron activity directly in vivo has now circumvented many of these limitations^6^. Using GCaMP photometry, we show that ovariectomy results in dramatic estrogen-dependent changes to the activity of the GnRH pulse generator. Remarkably, given the myriad direct and indirect mechanisms potentially involved in this pathway^3,4,19,31–33^, the estrogen-dependent plasticity of ARN^KISS^ neuron activity appears to be determined by a single gene *Esr1*. Genetic deletion of *Esr1* in all kisspeptin neurons generates the exact profile of ARN^KISS^ neuron hyperactivation found in OVX wild-type mice and this is not modified by OVX or estrogen administration. Further, in vivo CRISPR knockdown of ESR1 selectively in adult ARN^KISS^ neurons can generate the same OVX synchronization profile. We find that sufficient knockdown of ESR1 in ARN^KISS^ neurons results in a phenotype in which enhanced GnRH pulse generator activity operates through a dysfunctional pituitary to generate frequent low amplitude LH pulses. This serves to highlight the difficulties of examining negative feedback mechanisms when assessing only LH secretion, and also emphasizes the important equilibrium maintained by the GnRH pulse generator and the pituitary in generating pulsatile LH secretion.

We note that the frequency, duration, and amplitude of ARN^KISS^ neuron SEs all increase robustly following ovariectomy but do so over different time scales; the first changes are those of increased frequency and the appearance of clusters of events, with more gradual increases in the SE duration and amplitude occurring after one week. This suggests that diverse ESR1-dependent mechanisms drive ARN^KISS^ neuron plasticity. Prior studies have suggested that classic as well as non-classical ESR1 genomic mechanisms may be involved in the estrogen negative feedback mechanism^34^ and this may well involve ARN^KISS^ neurons^35^. The genes modulated by estradiol are beginning to be established in ARN^KISS^ neurons^36^ and include the neuropeptides *Kiss1*, *Tac2*, and *Pdyn* as well as those encoding T-type calcium channels, hyperpolarization-activated currents and *Slc17a6*^13,14,31,35,37^. Indeed, there is very clear evidence that ESR1 is critical for estrogen to down-regulate *Kiss1* mRNA expression in ARN^KISS^ neurons^13,14,35^. Electrophysiological studies have reported largely inconsistent effects of estrogen on multiple intrinsic and extrinsic parameters of ARN^KISS^ neurons including glutamate and GABA post-synaptic currents^19–21,32,38,39^. Curiously, with one exception^20^, these studies report changes that would be predicted to increase ARN^KISS^ neuron excitability in the presence of estrogen. Clearly this is not the case in vivo and further investigation will be required to identify the key gene programs modulated by estrogen to sculpt functional plasticity in ARN^KISS^ neurons.

We have employed a CRISPR knockdown approach to be able to target the ARN^KISS^ neuron population selectively and to achieve suppression of ESR1 in adulthood. While this provides many advantages over standard recombinase-mediated genetic strategies, it has its own caveats. Complete knockout is not achievable using this approach and, as result of the random nature of DNA repair following endonuclease cleavage, each animal exhibits a different degree of gene knockdown. While our initial studies found similar efficacy of gRNA-3 and −6 with respect to ESR1 knockdown in preoptic GABAergic neurons, this was not the case with ARN^KISS^ neurons where gRNA-6 was variable and overall, much less efficacious. Variable ESR1 knockdown was also achieved in the combined CRISPR/photometry study. Importantly, however, the range of ESR1 deletion achieved in these studies has highlighted that neuronal or whole animal phenotypes only exist when greater then 70–80% of ESR1 is deleted from the ARN^KISS^ neurons. Also, as the suppression of ESR1 generates increased activity, it is sufficient for only one side of the ARN to be modified to drive disrupted functional output. These acute CRISPR studies indicate that 20–30% of ESR1-expressing kisspeptin neurons are sufficient for the negative feedback mechanism to operate, emphasizing once again the high degree of functional redundancy embedded within the neural circuitry controlling fertility^40,41^.

It is pertinent to consider the reasons why prior studies have struggled to establish a role for ESR1 in ARN^KISS^ neurons in negative feedback. The KERKO mice exhibit a remarkable advancement of puberty onset with elevated LH levels that then gradually return to basal levels as adults^27^. This has been interpreted as resulting from the immediate loss of negative feedback in the peripubertal period with compensatory mechanisms then coming into play to normalize LH release as adults^27^. As estradiol does not impact upon ARN^KISS^ neuron hyperactivity in KERKO mice, it is possible that the maintenance of some degree of negative feedback on LH secretion in adults^13,14^ arises from sustained inhibitory actions of estradiol at the pituitary. A similar scenario involving maintained negative feedback at the pituitary may explain results in rats with toxin-ablation of most ARN^KISS^ neurons^15^. These studies suggest that substantial plasticity may exist in the balance of estradiol negative feedback occurring at the gonadotroph and ARN^KISS^ neuron to enable relatively normal LH secretion. Finally, the high percentage of ESR1 deletion required to modify LH secretion reported here very likely explains the lack of effect of CRISPR-mediated ESR1 knockdown in ARN^KISS^ neurons on LH secretion in a prior study that achieved only 60% ESR1 suppression^16^.

The other key gonadal steroid involved in negative feedback is progesterone. Whereas estradiol is considered to provide a relatively constant suppression of pulse generator activity across the cycle, the high levels of progesterone secretion following ovulation are thought to provide an additional transient suppression that slows LH pulsatility during the ensuing estrus/luteal phase^42,43^. As ARN^KISS^ neurons express progesterone receptors^36^, it is possible that, like estradiol, progesterone acts directly on these neurons to slow their synchronization frequency. The genetic deletion of PR from all kisspeptin neurons results in abnormal fertility, involving principally anovulation^44^, but it has not yet been possible to examine the effects of ARN-selective PR deletion in kisspeptin neurons.

One intriguing observation has been that estrous cyclicity remained normal in ARN^KISS^ neuron ESR1 knockdown CRISPR mice with abnormal high frequency, low amplitude LH pulses. This indicates that this pattern of LH release is sufficient for normal periodic ovarian function and circulating estradiol levels. We hypothesize that gonadotroph secretory capacity is set by on-going estradiol negative feedback as well as the rate of pulsatile exposure to GnRH. In the absence of estradiol in OVX mice, the pituitary is able to faithfully “transmit” a ~20 min interval GnRH stimulus. In contrast, when the pituitary alone remains under estradiol negative feedback, as in CRISPR mice, the same ~20 min interval GnRH stimulus from hyperactive kisspeptin neurons is only transmitted as erratic small amplitude LH pulses. Albeit in a very unphysiological setting, this down regulation in gonadotroph sensitivity to GnRH may buffer ARN^KISS^ neuron hyperactivity to help preserve reproductive competency.

The dramatic, estrogen-dependent re-modeling of GnRH pulse generator activity is reminiscent of ARN multi-unit recordings obtained from OVX female monkeys^45,46^ suggesting that it is a conserved feature of mammalian reproductive networks. The appearance of SE clusters is particularly intriguing and appears to resemble a highly unstable state of ARN^KISS^ neuron activity with repeated events occurring immediately after one another for up to 15 min before terminating. While this excessive pattern of ARN^KISS^ neuron activation may well contribute to the development of perimenopausal hot flushes^47^ its impact on LH secretion is unclear. Unfortunately, presumably due to stress, these SE clusters are almost always reduced to a single SE event when undertaking tail-tip blood sampling and resulted in only a very limited evaluation of the relationship of SE clusters to pulsatile LH secretion. Nevertheless, we observed that an LH pulse was only associated with the first SE event within a cluster. This is similar to the relationship between cluster-like MUA events in the monkey ARN and pulsatile LH secretion^48^.

Experiments over many years have implicated a wide variety of mechanisms as contributing to the estrogen negative feedback mechanism. These have ranged from direct estrogen actions at the GnRH neuron to estrogen modulation by multiple different afferent populations and actions at non-neuronal cells within the network^3,4,33,49,50^. The challenge has been to establish a functional hierarchy amongst the various possible mechanisms for negative feedback. For example, there has long been interest in an estrogen-modulated GABAergic input to the GnRH neuron cell body as being involved in negative feedback^3,33^. However, recent studies have shown that estrogen negative feedback is normal in mice with global GABA neuron-selective ESR1 deletion^26^ and, more broadly, a role for neural modulation of the GnRH neuron cell body in regulating pulsatile LH secretion is now doubtful^42,51^. Remarkably, we found that ESR1 expressed by ARN^KISS^ neurons accounts for all the negative feedback actions of estrogen on the GnRH pulse generator. This demonstrates that estrogen modulation of the ARN^KISS^ neuron input to the GnRH neuron is the principal pathway underlying the estrogen negative feedback of pulsatile GnRH secretion in mice. Thus, alongside important effects of estradiol occurring at the pituitary, the estrogen negative feedback control of LH secretion is primarily achieved through direct ESR1-dependent modulation of ARN^KISS^ neurons.

## Methods

### Animals

*Kiss1-Cre;tdT* mice were generated by crossing 129S6Sv/Ev C57BL6 *Kiss1-Cre* mice^52^ with the C57BL/6 J Ai9-CAG-tdTom^+/−^ reporter line (JAX stock #07909)^53^ as described and characterized previously^6,54^. Kiss1-selective ESR1 knockout (KERKO) mice were generated by crossing *Kiss1*-*Cre;tdT* mice with a well characterized C57BL/6 line in which exon 3 of *Esr1* is floxed^55,56^. *Kiss1-Cre;LSL-Cas9-EGFP* mice were generated by crossing the *Kiss1-Cre* mice with B6J.129(B6N) *Rosa26-LSL-Cas9-EGFP* mice (JAX stock #026175)^28^. Mice used for photometry were individually housed in open top cages for the duration of the experiments. All mice were provided with environmental enrichment under conditions of controlled temperature (22 ± 2 °C), humidity (40–70%), and lighting (12 h light/12 h dark cycle; lights on at 6:00 h and off at 18:00 h) with ad libitum access to food (Teklad Global 18% Protein Rodent Diet 2918, Envigo, Huntingdon, UK) and water. Daily vaginal cytology was used to monitor the estrous cycle stage. All animal experimental protocols were approved by the Animal Welfare Committee of the University of Otago, New Zealand (96/2017) or the UK Home Office (P174441DE) for work at the University of Cambridge.

### Stereotaxic surgery and injections

Adult mice (3–4 months old) were anaesthetized with 2% Isoflurane, given local Lidocaine (4 mg/kg, s.c.) and Carprofen (5 mg/kg, s.c.) and placed in a stereotaxic apparatus. A custom-made bilateral Hamilton syringe apparatus holding two 25- or 29-gauge needles 0.9 mm apart was used to perform bilateral injections into the ARN. The needles were lowered into place over 2 min and left in situ for 3 min before the injection was made. The AAV was injected into the ARN of at a rate of ~100 nl/min with the needles left in situ for 10 min before being withdrawn. Carprofen (5 mg/kg body weight, s.c.) was administered for post-operative pain relief.

For CRISPR knockdown, bilateral injections of 1.5 μL AAV1-U6-gRNA-LacZ/ESR1-3/ESR1-6-Ef1α-mCherry-WPRE-SV40 (1.3–2.5 × 10^13^ GC/mL) were given into the ARN and mice allowed to recover for 3 weeks before commencing the experimental protocol.

For standard photometry experiments, mice received a 1 μL AAV9-CAG-FLEX-GCaMP6s-WPRE-SV40 (1.3 × 10^13^ GC/mL, University of Pennsylvania Vector Core) injection into the ARN followed by implantation of a unilateral indwelling optical fiber (400 µm diameter; 0.48 NA, Doric Lenses, Quebec, Canada) positioned directly above the mid-caudal ARN using the coordinates AP −1.2, DV −5.8. After surgery, mice received daily handling and habituation to the photometry recording procedure over 4–6 weeks before experimentation.

For CRISPR-photometry experiments, mice were given the same GCaMP6 AAV injection but implanted at the same time with a unilateral “fluidic” optic fiber consisting of a 400 µm diameter, 0.48 NA optic fiber combined with a fluid injection port (Doric Lenses, Quebec, Canada). After habituation and baseline recording, 1 μL AAV1-U6-gRNA(3/LacZ)-Ef1α-mCherry-WPRE-SV40 gRNA was delivered down the injection port over a period of 10 min.

### GCaMP6 fiber photometry

Photometry was performed as reported previously^6,24^. Fluorescence signals were acquired using a custom-built fiber photometry system made primarily from Doric components based on a previous design^57^. Violet (405 nm) and blue (465–490 nm) fiber-coupled LEDs were sinusoidally modulated at 531 and 211 Hz, respectively, and focused into a 400μm-diameter optic fiber which connected to the mouse. Emitted fluorescence was collected by the same fiber, passed through a 500–550 nm emission filter, and focused onto a photoreceiver (2151 Newport). The two GCaMP6s emissions were collected at 10 Hz in a scheduled 5 s on/15 s off mode by demodulating the 405 nm (non-calcium dependent) and 490 nm (calcium dependent) signals. The power output at the tip of the fiber was set at 50μW. Fluorescence signals (490-405) were collected and converted to ΔF/F (%) values as follows: ΔF/F = 100 × (F − Fb)/F where Fb was the basal fluorescence signal between events and F the recorded fluorescence.

All recordings were obtained from freely behaving mice between 09:00 and 13:00 h. Intact animals were recorded for 2 h in the diestrous stage of the cycle. Following ovariectomy (as below), subsequent 2 h recordings were undertaken at 2, 4, 8, 16, and 32 days. For the E2 replacements studies, OVX mice were re-recorded and then given an E2 capsule (as below) and further recordings undertaken 3 and 7 days later. The same procedure was undertaken for KERKO mice.

Synchronization events (SEs) were defined as abrupt peaks in ΔF/F > 10% of maximum signal strength. The between animal variability in signal means that changes in SE amplitude can only be reported as relative changes within an animal across recordings with the mean amplitude of SEs in the first recording set at 1.0. As OVX mice exhibit clusters of SEs, we defined the start of each cluster as a synchronization initiation (SI) with the number of on-going SEs determining whether this was a singlet, doublet, triplet, or quadruplet cluster (Fig. 1a). The frequency of SIs was calculated by determining the total number of SIs occurring during the 2 h recording period. The SI interval was determined by averaging the intervals between SIs or, where only a single SI occurred in the 2 h period (as can happen in intact and OVX E2-treated mice), the longest of the interval between the SI and the start or end of the recording was used. The total duration of an SE cluster was determined by measuring the mean time from the initial increase to return to basal fluorescence while the intracluster SE interval was the mean of all intervals between SEs within each cluster.

### Ovariectomy, estrogen replacement, and pulsatile LH assay

Bilateral ovariectomy was performed under Isoflurane anesthesia with pre- and post-operative Carprofen (5 mg/kg body weight, s.c.). Estradiol replacement was provided by s.c. implantation of an ~1 cm length of Silastic capsule (Dow Corning, USA) filled with 0.4 µg/ml 17-β-estradiol to provide 4 μg 17-β-estradiol/20 g body weight. This protocol returns the plasma profile of pulsatile LH secretion and 17-β-estradiol concentrations to that found in diestrous females^25^. Pulsatile LH secretion was assessed using the tail-tip bleeding methodology and ultrasensitive LH ELISA of Steyn and colleagues^29,30^. Combined GCaMP fiber photometry and 5 min interval tail-tip blood sampling (3 μL) was undertaken as reported previously^6,24,58^. The LH ELISA had an assay sensitivity of 0.04 ng/mL and intra-and inter-assay coefficients of variation of 4.6% and 9.3%. Pulse analysis was undertaken with PULSAR Otago^25^ using the following validated parameters: smoothing 0.7, peak split 2.5, level of detection 0.04, amplitude distance 3 or 4, assay variability 0 2.5 3.3, G values of 3.5,2.6,1.9,1.5,1.2 (intact) and 2.2, 2.7, 1.9, 1.5, 1.2 (OVX).

### CRISPR gRNAs and evaluation in vitro

Six gRNAs (Supplementary Fig. 1) were ordered from GenScript cloned into plasmid PX552. Plasmids for AAV production were prepared by transforming OneShot™TOP10 Chemically Competent *E.coli* (ThermoFisher Scientific). Transformed bacteria were cultured in LB broth supplemented with 100 μg/mL ampicillin and plasmids subsequently prepared using PureLink™ HiPure plasmid midiprep kit (ThermoFisher Scientific). Quantification of DNA was performed using NanoDrop (MaestroNano) and quality verified by agarose gel analysis.

Viral particle production was achieved using 293AAV cells (Cell Biolabs Inc., AAV-100) cultured in DMEM High Glucose with 10% FBS and 1% penicillin/streptomycin to 80–90% confluence before co-transfection with pAAV-DJ, pAAV-Helper and gRNA1-6 PX552 (all plasmids 6 µg/T75cm^2^ flask) in a 1:1 ratio using Lipofectamine 3000 reagent (Invitrogen) in DMEM High Glucose only. Transfected cells were cultured under standard cell culture conditions (21% O_2_, 5% CO_2_, 37 °C humified air) for 72 h, after which AAVs were purified from the cell culture supernatant using the Virabind AAV purification kit (Cell Biolabs) and AAV titer determined by SYBRGreen-based qPCR.

To generate an ESR1-positive cell line stably expressing Cas9, mHypoA2/29Clu189 cells (CELLutions Biosystems, Ontario, Canada) were cultured to 80–90% confluence and transfected with pSpCas9(BB)−2A-Puro (PX459, Addgene) using Lipofectamine 3000 (Invitrogen). After 18 days, four subclones (A-D) were plated into T25cm^2^ flasks from six-well culture plates, cultured to 80–90% confluence, and tested for ESR1 and Cas9 expression using western blot. All four clones expressed both proteins and Clone C was randomly chosen to subclone further. Thirty-five single cells from Clone C were each seeded into a single well of a 96-well cell culture plate and cultured in DMEM High Glucose, 10% FBS, 1% penicillin/streptomycin, and 7.5 μg/mL puromycin for 3 weeks. Subclones C1-35 were tested for Cas9 expression using Western blot analysis with Clone 22 showing the strongest expression.

CLU189Cas-9C22 cells were seeded at 1 × 10^5^ cells/well in a six-well dish overnight. 10% Virabind (Cell Biolabs) was added to the media and cells incubated overnight before transduction the next day with 1 μL of gRNA AAVs (~1 × 10^13^ GC/mL) per mL of cell culture media. Cells were incubated under standard cell culture conditions for 96 h before total RNA was extracted using TRIZOL reagent (Invitrogen).

Western blots for ESR1 and Cas9 were undertaken on lysed cell contents transferred to PVDF membranes (1 hour, 100 V, 4 °C), blocked in TBST buffer (10 mM Tris pH 7.5, 100 mM NaCl, 0.05% Tween 20) with 5% non-fat dry milk powder for 1 h at room temperature, and incubated overnight (4 °C) in polyclonal rabbit anti-ESR1 (1:1000, #06-935, Merck-Millipore, USA) or monoclonal anti-FlagM2 tagged to Cas9 (1:1000, #F1804, Sigma-Aldrich) in TBST buffer. Membranes were washed and incubated in goat anti-rabbit IgG horse radish peroxidase (HRP, Abcam ab97051) diluted 1:10,000 in TBST for ESR1 and goat anti-mouse IgG HRP (Abcam ab97023) for FlagM2 before bound HRP was detected using Pierce SuperSignal West Pico Chemiluminescent Substrate (ThermoFisher Scientific).

RTqPCR for *Esr1* was performed using Superscript III with 1 μg total RNA and oligodTs as per the recommended protocol (Invitrogen). qPCR was performed using 2 μL cDNA combined with Applied Biosystems TaqMan Fast Advanced Mastermix (ThermoFisher Scientific) and TaqMan Gene Expression Assay for *Esr1* (Mm00433149) or for Actb (Mm02619580). The delta delta C_T_ method was used to determine relative *Esr1* expression, using parental CLU189 cells as baseline controls.

For use with the Cas9/EGFP mouse line in vivo, the DNA cassette harboring the U6 promoter, gRNA, and scaffold sequence was PCR amplified from the PX552 vector and subcloned into pAAV-EF1a-mCherry (AddGene #1144770) using standard cloning procedures.

### In vivo CRISPR gRNA studies

The estrous cycles of adult female *Kiss1-Cre;LSL-Cas9-EGFP* mice were assessed for 3 weeks and mice exhibiting regular 4–6-day cycles given bilateral stereotaxic injections (1.5 μL) of AAV1-U6-gRNA-LacZ/ESR1-3/ESR1-6-Ef1α-mCherry into the ARN using the coordinates AP −1.2, DV −5.8. Three weeks later, estrous cycles were again monitored for 3 weeks. Pulsatile LH secretion was assessed using 6 min tail-tip bleeding for 180 min for intact mice (as above). At least 1 week later, diestrous-stage mice were given a GnRH stimulation protocol that involved taking a baseline tail-tip blood sample (3 µL), followed by s.c. 200 ng/kg GnRH (Bachem, Switzerland) followed by tail-tip blood samples taken 10 and 20 min later and analyzed for LH. Mice were then bilaterally ovariectomized as above and 2 weeks later underwent 4 min tail-tip bleeding for 120 min to assess pulsatile LH secretion. Mice were then anesthetized and killed for histological analysis (below).

For CRISPR-photometry studies, GCaMP photometry recordings were made from AAV-GCaMP6-injected *Kiss1-Cre;LSL-Cas9-EGFP* mice during diestrus as detailed above and then, under brief anesthesia, 1 μL of AAV1-U6-gRNA-LacZ or AAV1-U6-gRNA-ESR1-3 infused down the injection cannula integrated into the unilateral optic fiber. Photometry recordings commenced 3 weeks later.

### Immunohistochemistry

Mice were given a lethal overdose of pentobarbital (3 mg/100 μl, i.p.) and perfused through the heart with 20 mL of 4% paraformaldehyde in phosphate-buffered saline. Two or three sets of coronal sections (30 μm, 50 μm for CRISPR/photometry) were cut through the full extent of the ARN to assess optical fiber placements and expression of ESR1 in kisspeptin neurons. For KERKO experiments, brain sections from *Kiss1-Cre;tdT* with wild-type or null *Esr1* were processed for ESR1 using a well characterized rabbit antiserum^26^ (1:1000; #06-935, Merck-Millipore, USA) followed by biotinylated goat anti-rabbit immunoglobulins (1:400, Vector Laboratories) and streptavidin-conjugated 488 (1:200, Molecular Probes, USA). For in vivo CRISPR experiments, brain sections were incubated in a cocktail of chicken anti-EGFP^59^ (1:5000; AB13970, Abcam) and rabbit anti-ESR1 (as above) followed by goat anti-chicken 488 (1:200; Molecular Probes) and biotinylated goat anti-rabbit immunoglobulins (1:400, Vector Laboratories) and then Streptavidin 647 (1:200, Molecular Probes). This was followed by labeling for mCherry with rabbit anti-mCherry^59^ (1:10,000; Ab167453, Abcam) and goat anti-rabbit 568 (1;200; Molecular Probes). To assess ESR1 expression in TH-immunoreactive neurons, dual-label chromogen immunohistochemistry was undertaken with rabbit anti-ESR1 (1:5000), biotinylated goat anti-rabbit immunoglobulins (1:400, Vector Labs), and Vector Elite avidin-peroxidase (1:100) revealed with nickel-DAB followed by polyclonal rabbit anti-TH^60^ (1:5000; Chemicon AB152, RRID:AB_390204, Merck-Millipore) and peroxidase-labeled goat anti-rabbit (1:200, Vector Labs) with DAB as the chromogen. For CRISPR-photometry experiments, dual-label immunofluorescence was undertaken on slide-mounted sections to maintain tissue integrity using a cocktail of antisera against ESR1 (1:500) and chicken anti-EGFP (1:2500, Abcam) in 150 μL of incubation buffer placed on the slide in a humidified chamber at 4 °C for 40 h followed by biotinylated goat anti-rabbit immunoglobulins (1;200, Vector) and goat anti-chicken 488 (1:200, Molecular Probes) and then streptavidin-conjugated 647 (1:200, Molecular Probes) at room temperature for 90 min.

Quantitative analyses of ESR1 expression in ARN^KISS^ neurons were undertaken on confocal images captured on a Nikon A1R multi-photon laser scanning microscope using ×40 Plan Fluor, N.A. 0.75 objective using, software Nikon Elements C (v 3.22). The numbers of tdT- or EGFP-labeled cells with and without immunoreactive ESR1 nuclei were counted by an investigator blind to the experimental groupings. Cell counts were undertaken by analyzing all EGFP- or tdT-positive cells through 10 z-slices of 2 µm thickness in two sections at each of the rostral, middle and caudal levels of the ARN for each mouse. The number of TH neurons with ESR1 was assessed under brightfield microscopy by counting the number of TH-immunoreactive cells (brown DAB) with and without black (nickel-DAB) ESR1-positive nuclei in 2–3 sections at the level of the middle ARN in each mouse.

### Statistical analysis

Statistical analysis was undertaken on Prism 10 using repeated measures or one-way ANOVA with post hoc Dunnett’s multiple comparisons and Mann–Whitney U-tests where appropriate and as indicated. All tests were two-sided. Data are presented as mean ± SEM.

### Reporting summary

Further information on research design is available in the Nature Portfolio Reporting Summary linked to this article.

## Supplementary information

Supplementary Information

Reporting Summary

## Data Availability

All data generated or analyzed during this study are included in this published article and its supplementary information files, and are available from the corresponding author upon request. Source data are provided with this paper.

## Source data

Source Data
