# Supplementary material for: Definition of the estrogen negative feedback pathway controlling the GnRH pulse generator in female mice
Source: Nat Commun. 2022 Dec 2;13:7433. doi: 10.1038/s41467-022-35243-z (PMC9718805; doi:10.1038/s41467-022-35243-z)
Supplement: Supplementary file 1 — Supplementary Information [file 41467_2022_35243_MOESM1_ESM.pdf]

Supplementary data

Definition of the estrogen negative feedback pathway controlling the GnRH pulse generator in female mice

H. James McQuillan[1,2]*, Jenny Clarkson[1,2]*, Alexia Kauff[2], Su Young Han[1,2,3], Siew Hoong Yip[1,2], Isaiah Cheong[1,2], Robert Porteous[1,2], Alison K. Heather[2], Allan E. Herbison[1,2,3]

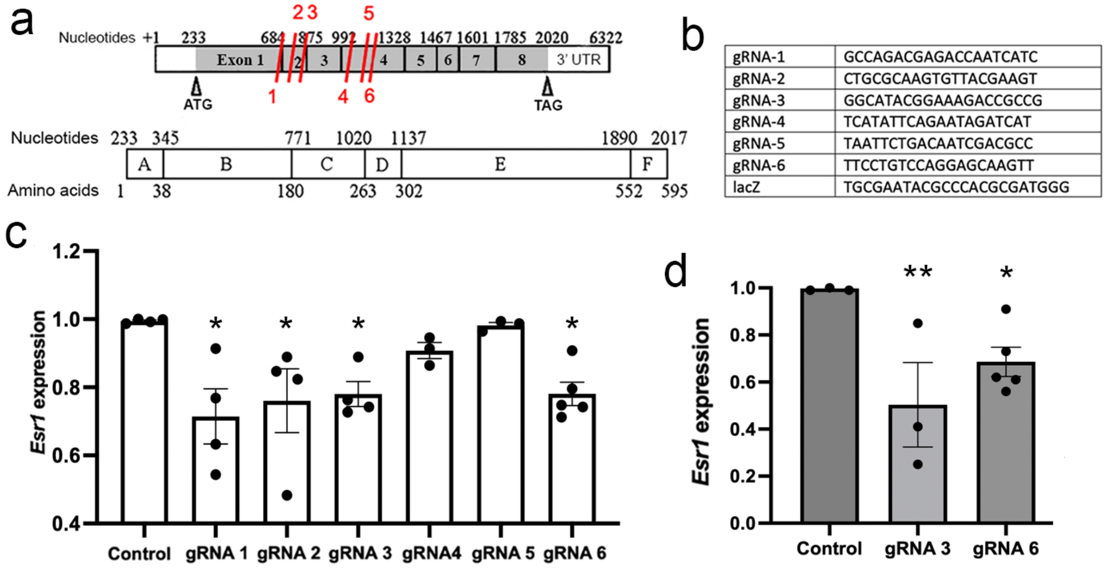

Suppl. Fig.1. *Design and in vitro evaluation of ESR1 CRISPR gRNAs*. **a,** Nucleotide and amino acid map of ESR1 showing the regions targeted for endonuclease cleavage by the six guide RNAS operating on the sense (red numbers above) or antisense DNA strand (red numbers below). **b,** gRNA sequences. **c,** Effects of 48 h treatment of the six gRNAs (AAV-U6-gRNA1-6-EGFP) on mean (± SEM) *Esr1* mRNA levels in ESR1-, Cas9- expressing cell line (CLU189-Cas922C) measured by RTqPCR, relative to *Actb.* Data is shown as ratio to parent cells, CLU-189 (control) (n=3 independent experiments). * *P* = 0.007 (gRNA1), 0.026 (gRNA2), 0.046 (gRNA3), 0.034 (gRNA6; one-way ANOVA with Dunnett's post-hoc comparison). **d,** Effects of 96 h treatment of pAAV-U6-gRNA3/6-Ef1a-mCherry on mean (± SEM) *Esr1* mRNA levels in CLU189-Cas922C cells measured by RTqPCR, relative to *Actb.* Data is shown as ratio to parent cells, CLU-189 (control) (n=3 independent experiments). * *P* = 0.0342, ** *P* = 0.0047 (one-way ANOVA with Dunnett's post-hoc comparison).

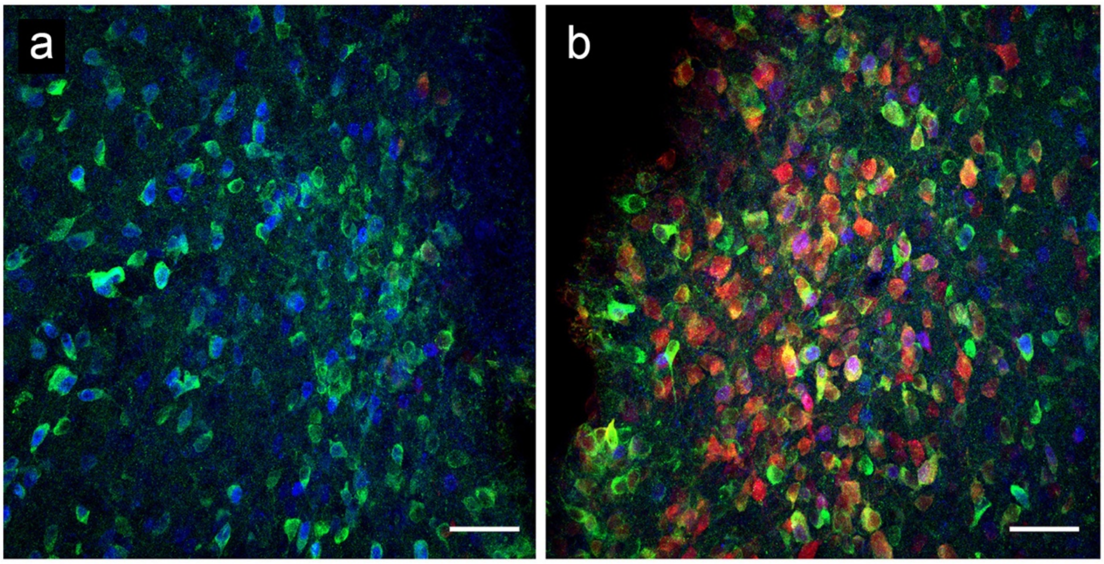

Suppl. Fig.2. *Crispr knockdown of ESR1 in medial preoptic area GABAergic neurons of Vgat-Cre,LSL-Cas9-EGFP*. **a,** Control un-injected side of the brain showing dual labelling for Vgat-targeted Cas9 (green EGFP) and ESR1 immunoreactivity (blue nuclei) in neurons. On average, 49±9% of EGFP-expressing neurons are positive for ESR1 (N=4). **b,** Other side of the brain injected with AAV1-U6-gRNA3-Ef1α-mCherry showing cells expressing EGFP/Cas9 and mCherry/gRNA (yellow-orange), mCherry alone (red), Cas9 alone (green) with or without ESR1 (blue nuclei). On average, 13±1% of EGFP-expressing neurons are positive for ESR1 (N=4). Scale bars represent 30 microns.

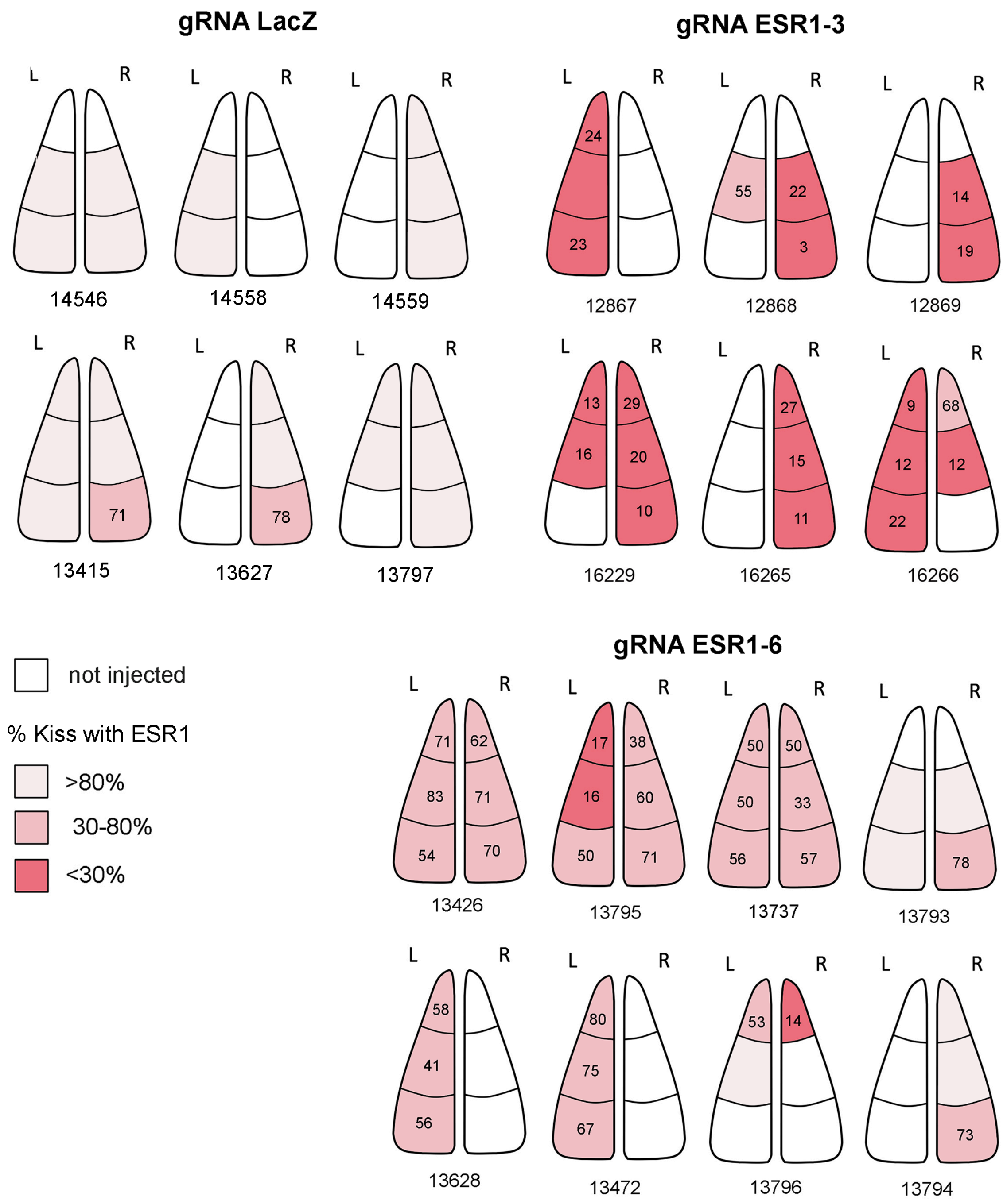

Suppl. Fig.3. *Distribution and degree of ESR1 knockdown in individual mice administered ESR1 gRNAs.* Flat maps show a birds-eye view of the ARN with the thin rostral pole extending to the wider caudal aspect for both sides of the ventricle (L and R). The animal number is shown below each flat map. The degree of ESR1 knockdown in ARN^{KISS} neurons is color-coded as indicated with the number in each segment representing the % kisspeptin neurons with ESR1 when co-expression was <80%. The middle ARN sections for animal 12867 were lost. Some mice received only unilateral injections as one of the bilateral injection cannulae was apparently blocked.

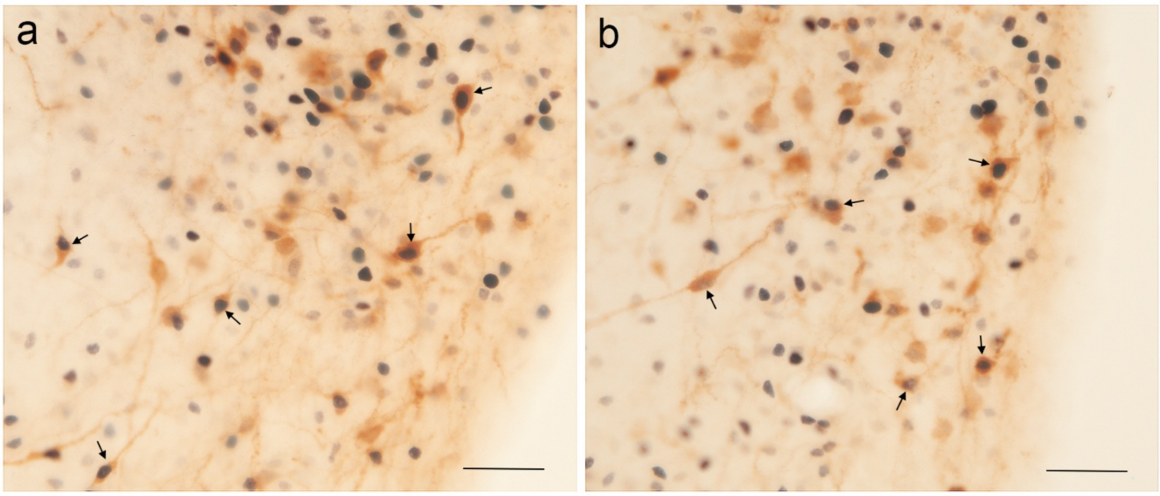

Suppl. Fig.4. *ESR1 expression in ARN dopamine neurons in Kiss1-ESR1 Crispr mice.* Dual-label immunohistochemistry showing ESR1 (black nickel-DAB nuclei) and tyrosine hydroxylase (brown DAB cytoplasm) in the ARN of *Kiss1-Cre,LSL-Cas9* mice injected with gRNA-lacZ (a) or gRNA-3 (b) into the ARN. Examples of dual-labelled cells are indicated with arrows. See Figure 5G for data from each mouse. Scale bars represent 50 μm.

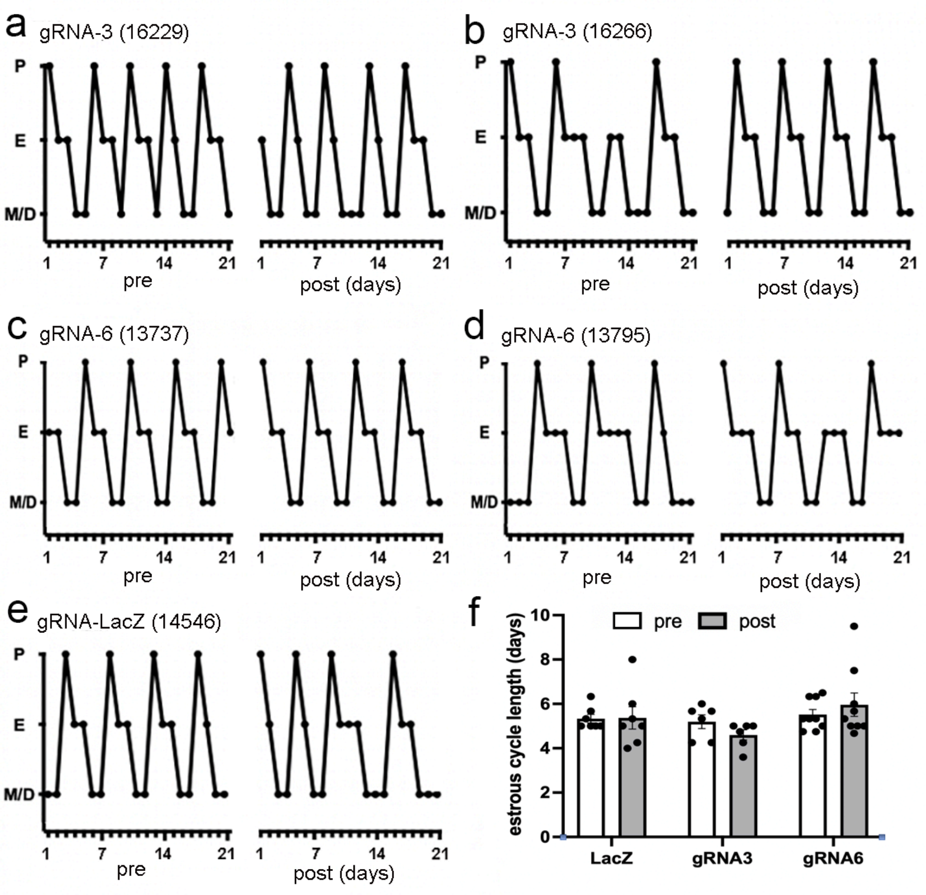

Suppl. Fig.5. *Estrous cyclicity in mice administered ESR1 gRNAs*.a-**e**, Estrous cycles are shown for representative gRNA-ESR1-3 (A,B), gRNA-ESR1-6 (C,D) and gRNA-LacZ (E) mice. **f,** Mean+SEM cycle length before and after gRNA injection in the three experimental groups (*N* = 6 to 9 in each experimental group).

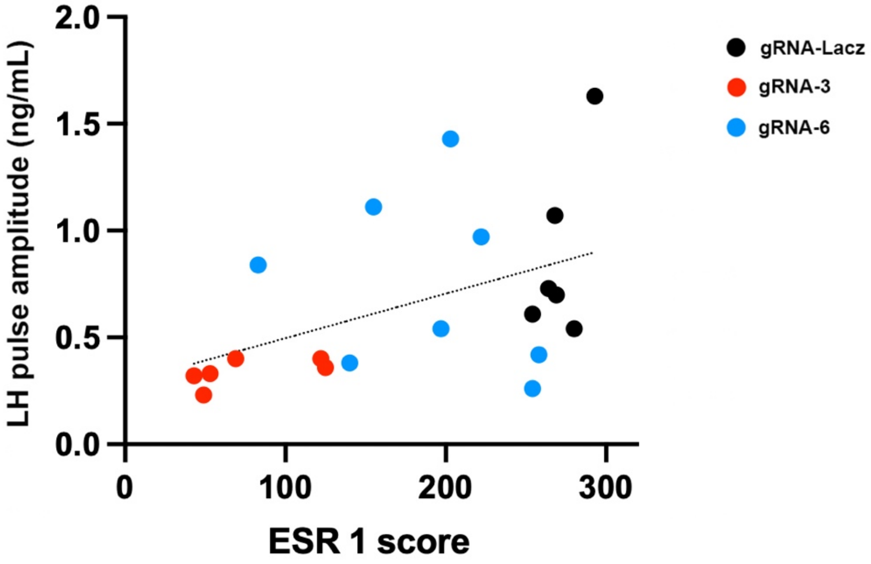

Suppl. Fig.6. *Correlation between overall ESR1 knockdown in ARN^{KISS} neurons and LH pulse amplitude in all mice*. Each color-coded dot shows the correlation between the mean amplitude of LH pulses detected by PULSAR in a mouse and an index of ESR1 expression (ESR1 score) by its ARN^{KISS} neurons. The ESR1 score was derived by combining the percentages of ARN^{KISS} neurons expressing ESR1 in the rostral, middle and caudal compartments of the ARN. To allow for mice with unilateral injections, the highest unilateral score was used for each mouse. Spearman correlation r = 0.5773, *P* = 0.0077.
