# Supplementary material for: Definition of the estrogen negative feedback pathway controlling the GnRH pulse generator in female mice
Source: Nat Commun. 2022 Dec 2;13:7433. doi: 10.1038/s41467-022-35243-z (PMC9718805; doi:10.1038/s41467-022-35243-z)
Supplement: Supplementary file 2 — Reporting Summary [file 41467_2022_35243_MOESM2_ESM.pdf]

nature portfolio

Corresponding author(s): Allan E. Herbison

Last updated by author(s): 27/10/22

Reporting Summary

Nature Portfolio wishes to improve the reproducibility of the work that we publish. This form provides structure for consistency and transparency in reporting. For further information on Nature Portfolio policies, see our Editorial Policies and the Editorial Policy Checklist.

Statistics

For all statistical analyses, confirm that the following items are present in the figure legend, table legend, main text, or Methods section.

n/a	Confirmed	
☐	☒	The exact sample size (*n*) for each experimental group/condition, given as a discrete number and unit of measurement
☐	☒	A statement on whether measurements were taken from distinct samples or whether the same sample was measured repeatedly
☐	☒	The statistical test(s) used AND whether they are one- or two-sided<br>*Only common tests should be described solely by name; describe more complex techniques in the Methods section.*
☒	☐	A description of all covariates tested
☐	☒	A description of any assumptions or corrections, such as tests of normality and adjustment for multiple comparisons
☐	☒	A full description of the statistical parameters including central tendency (e.g. means) or other basic estimates (e.g. regression coefficient) AND variation (e.g. standard deviation) or associated estimates of uncertainty (e.g. confidence intervals)
☐	☒	For null hypothesis testing, the test statistic (e.g. *F*, *t*, *r*) with confidence intervals, effect sizes, degrees of freedom and *P* value noted<br>*Give P values as exact values whenever suitable.*
☒	☐	For Bayesian analysis, information on the choice of priors and Markov chain Monte Carlo settings
☒	☐	For hierarchical and complex designs, identification of the appropriate level for tests and full reporting of outcomes
☒	☐	Estimates of effect sizes (e.g. Cohen's *d*, Pearson's *r*), indicating how they were calculated

Our web collection on statistics for biologists contains articles on many of the points above.

Software and code

Policy information about availability of computer code

Data collection	Open-source Neuromouse fibre photometry data acquisition system, developed by Tussock Innovation and Argotech (New Zealand). Utilizes Doric (Quebec, Canada) LED drivers and components with National Instrument DAQ board (USB-6211)<br>Nikon Elements C (v3.22)
Data analysis	Prism 10 statistical software

For manuscripts utilizing custom algorithms or software that are central to the research but not yet described in published literature, software must be made available to editors and reviewers. We strongly encourage code deposition in a community repository (e.g. GitHub). See the Nature Portfolio guidelines for submitting code & software for further information.

Data

Policy information about availability of data

All manuscripts must include a data availability statement. This statement should provide the following information, where applicable:

- Accession codes, unique identifiers, or web links for publicly available datasets
- A description of any restrictions on data availability
- For clinical datasets or third party data, please ensure that the statement adheres to our policy

All data generated or analysed during this study are included in this published article (and its supplementary information files). Source data are provided with this

paper.

Human research participants

Policy information about studies involving human research participants and Sex and Gender in Research.

Reporting on sex and gender	N/A
Population characteristics	N/A
Recruitment	N/A
Ethics oversight	N/A

Note that full information on the approval of the study protocol must also be provided in the manuscript.

Field-specific reporting

Please select the one below that is the best fit for your research. If you are not sure, read the appropriate sections before making your selection.

[x] Life sciences [ ] Behavioural & social sciences [ ] Ecological, evolutionary & environmental sciences

For a reference copy of the document with all sections, see nature.com/documents/nr-reporting-summary-flat.pdf

Life sciences study design

All studies must disclose on these points even when the disclosure is negative.

Sample size	Sample sizes were determined based upon convention in this field of research.
Data exclusions	Data from one mouse receiving gRNA-Lacz was excluded due to abnormally high LH levels (7.0 ng/mL) in the intact state.
Replication	Mice were analyzed in 3 or 4 separate mixed genotype and treatment cohorts with each replicating the same observation. All data is presented.
Randomization	Genotypes and gRNA treatments were randomized within each experimental cohort.
Blinding	Data collection and analysis was undertaken by investigators blinded to the treatment groups.

Reporting for specific materials, systems and methods

We require information from authors about some types of materials, experimental systems and methods used in many studies. Here, indicate whether each material, system or method listed is relevant to your study. If you are not sure if a list item applies to your research, read the appropriate section before selecting a response.

Materials & experimental systems

n/a	Involved in the study	
[ ]	[x]	Antibodies
[ ]	[x]	Eukaryotic cell lines
[x]	[ ]	Palaeontology and archaeology
[ ]	[x]	Animals and other organisms
[x]	[ ]	Clinical data
[x]	[ ]	Dual use research of concern

Methods

n/a	Involved in the study	
[x]	[ ]	ChIP-seq
[x]	[ ]	Flow cytometry
[x]	[ ]	MRI-based neuroimaging

Antibodies

Antibodies used	rabbit anti-TH 1:5,000; AB152, Merck-Millipore, USA<br>rabbit anti-ESR1 1:1,000; #06-935, Merck-Millipore, USA

	chicken anti-EGFP 1:5,000; AB13970, Abcam, UK<br>rabbit anti-mCherry 1:10,000; Ab167453, Abcam, UK
Validation	Citations describing the specificity of each of the above antisera for use in the mouse brain are provided in the Methods section of the text.

Eukaryotic cell lines

Policy information about cell lines and Sex and Gender in Research

Cell line source(s)	An immortalized male mouse hypothalamic cell line expressing ESR1 (mHypoA2/29Clu189 cells) was obtained from a Dr. Alex Tupps (Univeristy of Otago) and are available from CELLutions Biosystems, Canada.
Authentication	The expression of ESR1 was authenticated using RT-PCR.
Mycoplasma contamination	The cell line was tested negative for Mycoplasma.
Commonly misidentified lines (See ICLAC register)	None

Animals and other research organisms

Policy information about studies involving animals; ARRIVE guidelines recommended for reporting animal research, and Sex and Gender in Research

Laboratory animals	All mice were investigated when adult (older than postnatal day 65).<br>129S6Sv/Ev C57BL6 Kiss1-Cre mouse line (Prof. Bill Colledge, Cambridge, UK)<br>C57BL/6J Ai9-CAG-tdTom mouse line (JAX stock #07909)<br>C57BL/6 Esr1 flox line (Prof. Günther Schütz, Heildelberg, Germany)<br>B6J.129(B6N) Rosa26-LSL-Cas9-EGFP mouse line (JAX stock #026175)
Wild animals	Wild animals were not used in this study.
Reporting on sex	This study was deliberately designed to address females only as the estrogen negative feedback mechanism is responsible for the generation of ovarian/estrous cycles in female mammals.
Field-collected samples	The study did not involve field collected samples.
Ethics oversight	Experiments were approved and undertaken under the Animal Welfare Committee of the University of Otago, New Zealand (96/2017) or the UK Home Office (P174441DE) for work at the University of Cambridge.

Note that full information on the approval of the study protocol must also be provided in the manuscript.
